# A-series agent A-234: initial in vitro and in vivo characterization

**DOI:** 10.1007/s00204-024-03689-3

**Published:** 2024-03-06

**Authors:** Martina Hrabinova, Jaroslav Pejchal, Vendula Hepnarova, Lubica Muckova, Lucie Junova, Jakub Opravil, Jana Zdarova Karasova, Tomas Rozsypal, Alzbeta Dlabkova, Helena Rehulkova, Tomas Kucera, Zbyněk Vecera, Filip Caisberger, Monika Schmidt, Ondrej Soukup, Daniel Jun

**Affiliations:** 1 University of Defence, Military Faculty of Medicine, Department of Toxicology and Military Pharmacy, Trebesska 1575, 500 01 Hradec Králové, Czech Republic; 2 University Hospital Hradec Kralove, Biomedical Research Centre, Sokolska 581, 500 05 Hradec Králové, Czech Republic; 3 University of Defence, Nuclear, Biological, and Chemical Defence Institute, Vita Nejedleho 1, 68203 Vyskov, Czech Republic; 4 University of Defence, Military Faculty of Medicine, Department of Military Medical Service Organization and Management, Trebesska 1575, 500 01 Hradec Králové, Czech Republic; 5 University Hospital Hradec Kralove, Department of Neurology, Sokolska 581, 500 05 Hradec Králové, Czech Republic; 6 University Hradec Kralove, Department of Chemistry, Faculty of Science, Rokitanskeho 62, 50003 Hradec Králové, Czech Republic

**Keywords:** Nerve agent A-234, Hydrolysis, Reactivation, Acute toxicity, Therapy

## Abstract

A-series agent A-234 belongs to a new generation of nerve agents. The poisoning of a former Russian spy Sergei Skripal and his daughter in Salisbury, England, in March 2018 led to the inclusion of A-234 and other A-series agents into the Chemical Weapons Convention. Even though five years have already passed, there is still very little information on its chemical properties, biological activities, and treatment options with established antidotes. In this article, we first assessed A-234 stability in neutral pH for subsequent experiments. Then, we determined its inhibitory potential towards human recombinant acetylcholinesterase (*Hss*AChE; EC 3.1.1.7) and butyrylcholinesterase (*Hss*BChE; EC 3.1.1.8), the ability of HI-6, obidoxime, pralidoxime, methoxime, and trimedoxime to reactivate inhibited cholinesterases (ChEs), its toxicity in rats and therapeutic effects of different antidotal approaches. Finally, we utilized molecular dynamics to explain our findings. The results of spontaneous A-234 hydrolysis showed a slow process with a reaction rate displaying a triphasic course during the first 72 h (the residual concentration 86.2%). A-234 was found to be a potent inhibitor of both human ChEs (*Hss*AChE IC_50_ = 0.101 ± 0.003 µM and *Hss*BChE IC_50_ = 0.036 ± 0.002 µM), whereas the five marketed oximes have negligible reactivation ability toward A-234-inhibited *Hss*AChE and *Hss*BChE. The acute toxicity of A-234 is comparable to that of VX and in the context of therapy, atropine and diazepam effectively mitigate A-234 lethality. Even though oxime administration may induce minor improvements, selected oximes (HI-6 and methoxime) do not reactivate ChEs in vivo. Molecular dynamics implies that all marketed oximes are weak nucleophiles, which may explain the failure to reactivate the A-234 phosphorus-serine oxygen bond characterized by low partial charge, in particular, HI-6 and trimedoxime oxime oxygen may not be able to effectively approach the A-234 phosphorus, while pralidoxime displayed low interaction energy. This study is the first to provide essential experimental preclinical data on the A-234 compound.

**Supplementary Information:**

The online version contains supplementary material available at 10.1007/s00204-024-03689-3.

## Introduction

**Fig. 1:**
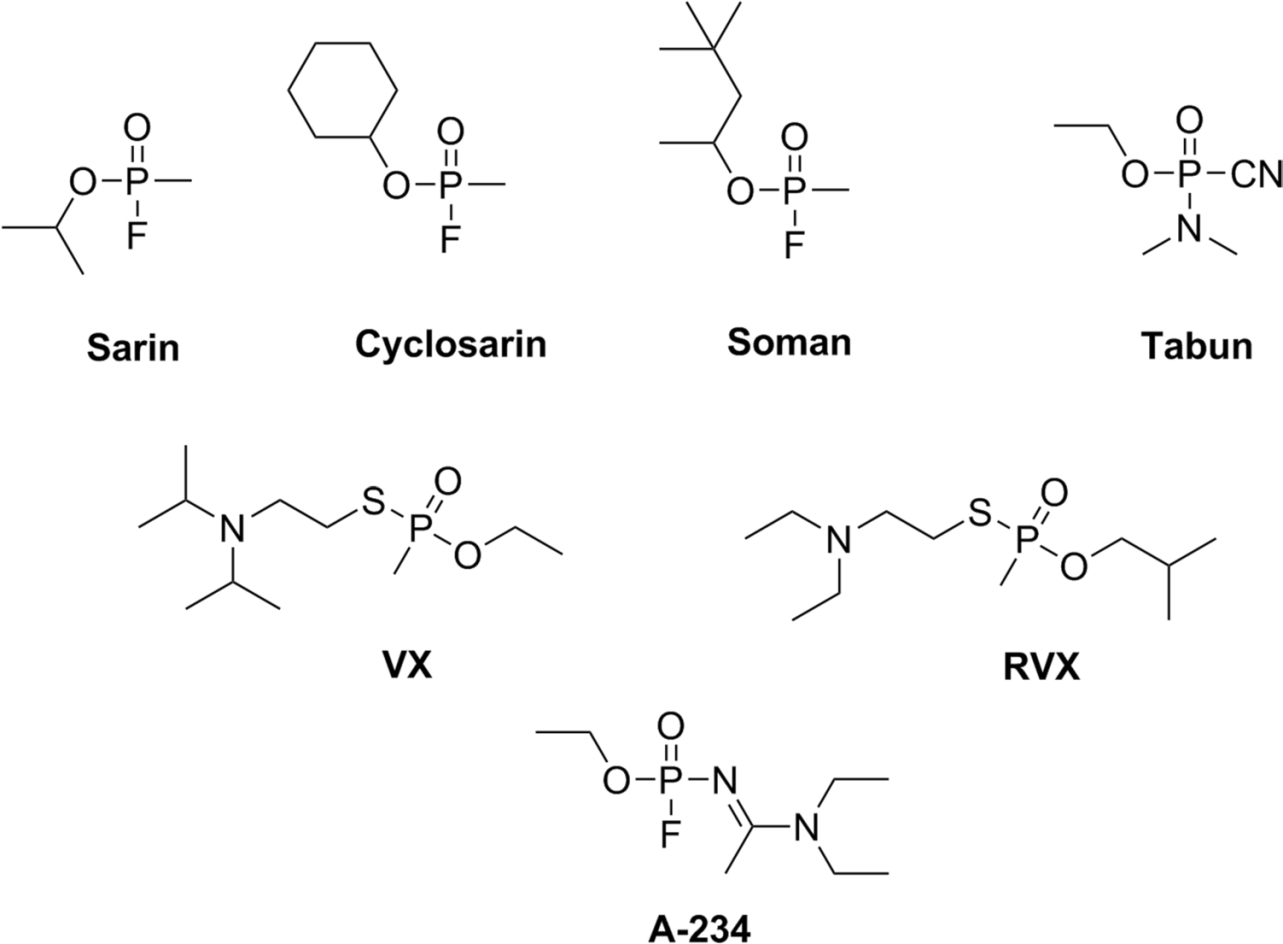
Structures of selected nerve agents

Organophosphates (OPs) were initially developed through pesticide research programs. However, despite the expected selectivity to other species, many newly synthesized substances showed high toxicity to humans. Indeed, the very first OP intoxication case published in the Archives of Toxicology was caused by parathion (Vogel 1953). Symptoms of intoxication involve increased salivation, lachrymation, diarrhea, nausea, vomiting, miosis, sweating, muscle tremors, seizures, confusion, or unconsciousness (Marrs 1993). Severe OP intoxication may lead to death, typically due to respiratory failure from the combination of bronchospasms, bronchorrhea, central respiratory depression, and respiratory muscle weakness/paralysis (Hulse et al. 2014). Military strategists soon realized the potential of these substances. The company IG Farben synthesized compounds designated as G-nerve agents as a part of a chemical weapon program between the 1930s and 1950s. Thus, tabun (GA), sarin (GB), soman (GD), and cyclosarin (GF) (Fig. 1) were discovered. They have a higher human acute toxicity than commonly used pesticides. With even higher toxicity, VX and VR (Fig. 1) were produced later during the Cold War in Great Britain and the Soviet Union, respectively. The latest A-series of the nerve agents was developed in the 1970s in the Soviet Union. The so-called Novichok agents were prepared as binary weapons and are claimed to be even more toxic than VX (Hrvat and Kovarik 2020) (for the detailed metaanalysis see Opravil et al. 2023). In March 2018, after the attempted murder of a former Russian spy and his daughter in Salisbury, UK, the term Novichok came to public attention. The British government stated that the nerve agent A-234 traces (Fig. 1) were found at the crime scene (Lewis 2018; Vogel et al. 2021). Due to this incident, the Organization for Prohibition of Chemical Weapons (OPCW) banned Novichok agents, placing them on Schedule I of the Chemical Weapons Convention (OPCW 2019). Later on, in August 2020, the compound from the Novichok family (without specification) was also used in the assassination attempt on a Russian opposition politician Alexei Navalny (OPCW 2020; Steindl et al. 2021).

However, several years after these cases, there is still insufficient reliable scientific information on Novichok substances. Although the first result articles have appeared recently, theoretical or in silico studies still dominate the scientific papers (Chai et al. 2018; Nepovimova and Kuca 2018; Jeong and Choi 2019; Bhakhoa et al. 2019; Franca et al. 2019; Imrit et al. 2020).

This manuscript extends general knowledge about compound A-234, ethyl *N*-[(1E)-1-(diethylamino)ethylidene]-phosphoramidofluoridate in terms of stability, AChE-inhibition potential, reactivation capability, in vivo acute toxicity, and countermeasures.

## Material and methods

### Hydrolysis of A-234

A 100 mL of the analyte of A-234 (batch purity 87.1% was quantified by GC-FID prior to the measurement; Nuclear, Biological, and Chemical Defence Institute, University of Defence, Vyskov, Czech Republic) at a concentration of 100 µg mL^−1^ in 0.05 M phosphate buffer (PBS, pH 7; Penta, Prague, Czech Republic) was prepared to determine the kinetic curve of spontaneous hydrolysis. An aliquot volume of 1 ml was periodically taken from the solution and extracted into 1,2-dichloroethane (DCE; Merck, Darmstadt, Germany). The extraction efficiency of A-234 from the buffer to DCE was verified by repeatedly creating 5 solutions of A-234 in buffer pH 7 of the same concentration and subsequent immediate extraction into DCE. Extraction was optimized to 5 min vortexing with a frequency of 800 rpm in Multi Reax automatic shaker (Heidolph, Schwabach, Germany). The volume ratio was 1/1 (PBS/DCE). The calibration curve of the analyte ranged from 10 to 70 µg mL^−1^.

Subsequently, the concentration of A-234 in the organic extract was monitored by gas chromatography using a flame ionization detector (GC-FID) Trace 1310 at room temperature within 72 h. The column was TG-5MS, 30 m × 0.32 mm × 0.50 µm (both Thermo Fisher Scientific, Waltham, MA, USA). The oven temperature program started at 80 °C (2 min hold), followed by a gradient ramp of 20 °C min^−1^ until 280 °C, where the temperature was held for.

2 min. The method was 15 min long in total. Injections proceeded in a volume of 1 µL using the hot syringe method. The injection port was set at 250 °C, the injection proceeded in split mode (1:20 ratio), and the purge flow was 5 mL min^−1^. A constant flow rate of 1.5 mL min^−1^ of helium was applied to the column. The detector temperature was 280 °C. FID gases were set to a flow rate of 350 mL min^−1^ (air) and 40 mL min^−1^ (hydrogen). Furthermore, make-up gas (nitrogen) was introduced into the system at 30 mL min^−1^. All gases were of 5.0 purity. Chromeleon 7.3 software (Thermo Fisher Scientific) was used for data acquisition. The observed rate constant of the pseudo-first-order reaction *k* was calculated according to Eq. 1. GraphPad Prism version 9.5.1 for Windows (GraphPad Software, San Diego, CA, USA) was used for data presentation.

1 $$c_t = c_0 \cdot e^{ - kt}$$

Equation 1 Decrease in A-234 concentration during spontaneous hydrolysis in phosphate buffer pH 7.

### Biochemical assay

#### Enzyme production and purification

The nucleotide sequences encoding *Hss*AChE and *Hss*BChE were obtained from Invitrogen Technologies (Thermo Fisher Scientific). The cholinesterase (ChE) genes, optimized for expression in Expi293™ cells, were cloned into pcDNA™3.4 TOPO^®^ expression vector (Thermo Fisher Scientific). The verified constructs were transformed into NEB^®^ 10-beta *E. coli* cells (New England Biolabs, Ipswich, MA, USA). The PureLink^®^ HiPure Plasmid Filter Midiprep Kit (Thermo Fisher Scientific) was used to isolate the plasmid DNA. The proteins, carrying a C-terminal hexa histidine tag, were produced for seven days as secreted proteins at 37 °C, 8% CO_2_ atmosphere, and orbital shaking at an optimal spinner speed of approximately 125 rpm. At the end of the expression, the cell culture supernatant for *Hss*AChE and *Hss*BChE was collected and directly subjected to purification steps. Both ChEs were desalted using Amicon^®^ Ultra 15 mL centrifugal devices with Ultracel^®^ filters with a molecular weight cut-off of 30,000 Daltons (Merck) and subsequently purified using Ni–NTA affinity chromatography (GE Healthcare, Chicago, IL, USA). The proteins were eluted from the resin with a concentration gradient of 25–250 mM imidazole in 20 mM TRIS buffer (pH 7.5, 150 mM NaCl; Merck) followed by buffer exchange step using Amicon^®^ Ultra 15 mL centrifugal devices (cut-off of 30,000 Daltons (Merck). The protein expression level, its enzymatic activity and efficacy of purification steps were checked using Ellman’s method (Ellman et al. 1961). The kinetic parameters of the recombinant enzymes were verified by Km (281 ± 27 µM and 256 ± 3 µM for AChE using acetylthiocholine iodide (ATChI) substrate and BChE using butyrylthiocholine iodide (BTChI) (both from Merck), respectively, and IC_50_ values of standard inhibitors, e.g., donepezil for *Hss*AChE and ethopropazine for *Hss*BChE (both from Merck), and successfully compared with published results. The purified enzymes were lyophilized and stored at − 80 °C for future use.

#### Cholinesterase inhibitory assay

Modified Ellman’s protocol was used to investigate the inhibitory ability and selectivity of nerve agents towards *Hss*AChE and *Hss*BChE (Ellman et al. 1961). Polystyrene Nunc 96-well microplates with a flat bottom shape (Thermo Fisher Scientific) were utilized. The assay was carried out in 0.1 M KH_2_PO_4_/K_2_HPO_4_ buffer, pH 7.4. Enzyme solutions were prepared at 2.0 units/mL in 2 mL aliquots. The assay medium (100 µL) consisted of 40 µL of 0.1 M phosphate buffer (pH 7.4), 20 µL of 0.01 M DTNB, 10 µL of the enzyme, 10 µL of OP solution, and 20 µL of 0.01 M (ATChI/BTChI). Solutions of A-234, GB, or VX (batch purity of 99.0%, 99.0%, and 93.5%, respectively, as quantified by GC–MS before the measurement; all organophosphates were provided by Military Research Institute, Brno, Czech Republic) in propan-2-ol at concentrations ranging from 10^–4^ to 10^–8^ M were pre-incubated for 5 × half-life of inhibition and then measured (final assay concentration 10^–5^–10^–9^ M). The stability of A-234 in propan-2-ol is described in the Supplementary information (Fig. S1). The half-life evaluation, i.e., the time when the enzyme activity is reduced by 50%, is described in the supplementary information (Fig. S2). The reaction was started by adding the substrate. The activity was determined by measuring the increase in absorbance at 412 nm at 37 °C at 2 min intervals. The obtained data were used to compute the % of inhibition (% *I*; Eq. 2). The graphs for kinetic curves and half-life calculation were done using GraphPad Prism version 9.5.1 for Windows.

2 $$\% I = \left( {1 - \frac{E - E_i }{E}} \right) \cdot 100$$

Equation 2 E indicates enzyme activity without inhibitor (control), and E_I_ denotes inhibited enzyme activity (test sample).

#### Determination of inhibition rate constants (***k***_***i***_)

Modified Ellman’s protocol was also utilized to determine inhibition rate constants (Ellman et al. 1961). For that, the assay medium (100 µL) described in the previous chapter (2.2.2) was used with two adjustments. (i) OP solutions of A-234, GB, or VX in propan-2-ol ranged from 10^–6^ to 10^–9^ M (final concentration) and (ii) the medium components were immediately mixed without preincubation. ATChI hydrolysis was continuously monitored for up to 30 min. The recorded curves were analyzed by non-linear regression analysis to calculate *k*_i_ according to Eq. 3 (Aurbek et al. 2006).

3 $$k_i = k_2 /K_D$$

Equation 3 The *k*_2_ was determined as the reciprocal value of the intercept on the ordinate and *K*_D_ as the reciprocal value of the intercept on the abscissa (Forsberg and Puu 1984). The kinetic data and graphs were calculated and created by GraphPad Prism version 10.1.0. for Windows.

#### Cholinesterase reactivation endpoint assay

The reactivation potency of the standard oximes was evaluated on *Hss*AChE and *Hss*BChE. The enzyme was inhibited by a solution of appropriate ChE inhibitor A-234, GB, or VX in propan-2-ol at 100 μM for 5 × half-life of inhibition (Fig. S1) and the excess inhibitor was removed using an octadecylsilane-bonded silica gel SPE cartridge (UCT, Bristol, PA, USA). The inhibited enzyme was incubated for 10 min with a solution of HI-6, obidoxime, pralidoxime, methoxime, and trimedoxime chlorides (all from the Department of Toxicology and Military Pharmacy, University of Defence, Hradec Kralove, Czech Republic) at the concentration of 10^–4^ M at 37 °C. The reaction was initiated by adding substrate ATChI for *Hss*AChE and BTChI for *Hss*BChE. The activity of ChE was then measured spectrophotometrically at 412 nm by the modified Ellman’s method (Ellman et al. 1961). Each concentration of reactivator was assayed in triplicate. The obtained data were used to compute the % of reactivation potency (% *R*; Eq. 4). The results were corrected for oximolysis and inhibition of *Hss*AChE/*Hss*BChE by the reactivator. The graphs of reactivation were generated using GraphPad Prism version 9.5.1.

4 $$R = \left( {1 - \frac{\Delta A_0 - \Delta A_r }{{\Delta A_0 - \Delta A_i }}} \right) \cdot 100$$

Equation 4 Δ*A*_0_ indicates the change in absorbance caused by intact ChE (phosphate buffer was used instead of ChE inhibitor solution), Δ*A*_i_ indicates the change in absorbance provided by cholinesterase-exposed inhibitors, and Δ*A*_r_ indicates the change in absorbance caused by inhibited ChE by OP incubated with reactivator solution.

#### Reactivation kinetics assessment

OP-inhibited *Hss*AChE was prepared by adding A-234, GB, or VX at IC_50_ (0.101 µM, 0.417 µM, or 0.027 µM, respectively) and incubated for 5 × half-life of inhibition (Table 1). The excess inhibitor was removed using an octadecylsilane-bonded silica gel SPE cartridge. Next, 10 µL of 0.1 M phosphate buffer (pH 7.4) or 10 µL of HI-6 or methoxime (0.25–0.005 mM final concentration) was transferred to a 96-well microplate followed by 180 µL of assay solution containing 13.3 mL of 0.1 M phosphate buffer (pH 7.4), 3.7 mL of M DTNB, and 2 mL of ATChI. The reaction was initiated by adding the OP-inhibited or propan-2-ol-treated *Hss*AChE (10 µL). The residual enzyme activity was continuously measured at 412 nm for 30 or 60 min. All experiments were run at 37 °C and were performed in triplicate. Activities were corrected for oximolysis. The pseudo-first-order reactivation rate constant (*k*_r_) and the dissociation constant (*K*_D_) were calculated by nonlinear regression analysis using GraphPad Prism version 10.0.1, and the reactivity constant (*k*_r2_) were calculated according to Eq. 5*k*_r_ /*K*_D_. (Worek et al. 2002).

5 $$k_{r2} = k_r /K_D$$

**Table 1:** IC_50_ values (in 5 × half-life) and inhibition half-lives of A-234, GB, and VX against *Hss*AChE and *Hss*BChE (mean ± SEM, *n* = 3)

Compound	*Hss*AChE IC_50_ (µM)	*Hss*BChE IC_50_ (µM)	*t*_1/2_ *Hss*AChE inhibition (min)	*t*_1/2_ *Hss*BChE inhibition (min)
A-234	0.101 ± 0.003	0.036 ± 0.002	5.78	17.0
GB	0.417 ± 0.015	0.615 ± 0.025	6.63	8.71
VX	0.027 ± 0.001	1.080 ± 0.060	8.65	7.04

The experiments were performed in triplicate

Equation 5 The *k*_r2_ was determined as quotient of the reactivation rate constant (*k*_r_) and the dissociation constant (*K*_D_). The kinetic data and graphs were calculated and created by GraphPad Prism version 10.1.0. for Windows.

### In vivo

#### Animals

Male albino Wistar rats aged 9–11 weeks, weighing 220–260 g from VELAZ (Prague, Czech Republic), were housed in polypropylene solid bottom cages in climate- and access-controlled rooms (22 ± 2 °C and 50 ± 10% relative humidity) with light from 07:00 to 19:00 h and access to standard food and tap water ad libitum. The rats were acclimatized in the laboratory vivarium for 10 days before the experiments commenced. Subsequently, they were divided into groups and handled under the supervision of the Faculty of Military Health Sciences Ethics Committee in Hradec Kralove, Czech Republic (animal studies were approved under Nos. MO 188588/2021-1457, MO 292251/2021-1457, MO 285807/2022-1457).

#### A-234 acute toxicity, evaluation of drugs/antidotes therapeutic efficacy

To evaluate the therapeutic efficacy of commonly used drugs and antidotes, A-234-poisoned animals were treated with atropine sulfate (10 mg/kg), diazepam (2.5 mg/kg; both from Merck), and methoxime chloride (22.1 mg/kg), obidoxime chloride (10.5 mg/kg), or HI-6 chloride (39 mg/kg) based on previously published data in the literature (Kuca et al. 2013). Atropine, its combination with diazepam, or atropine-diazepam combination with oximes were administered *i.m*. 1 min after the *i.m*. A-234 challenge. The toxicity induced by A-234 was evaluated by assessing its LD_50_ value and 95% confidence interval (CI) using probit regression analysis (SPSS Statistics version 26, IBM, Armonk, NY, USA) of death occurring within 24 h after its administration at three to five different doses with six animals per dose (Tallarida and Murray 1987). The antidote/treatment efficacy was expressed as a protective ratio (LD_50_ value of protected rats/LD_50_ value of unprotected rats). The differences between LD_50_ values were tested based on chi-squared parallelism of regression lines and relative median potency (RMP) with 95% confidence limits (SPSS Statistics version 26). The two groups were considered significantly different if the 95% confidence limits did not include 1.0 (Lei and Sun 2018).

#### Functional observatory battery (FOB)

The neuroprotective effects of selected countermeasures were further studied using the FOB. The protection was tested only for combinations with HI-6 and methoxime. Diazepam was omitted so that it could not affect the examination results by its sedative effect. The animals were challenged with A-234 at the dose corresponding to 90% of LD_50_. Therapeutic approaches were the same as in the previous in vivo therapeutic efficacy experiments.

The FOB is a standardized set of behavioral and neurophysiological observations developed as a non-invasive procedure to detect significant functional deficits related to the neurotoxic effects of the studied compounds (Kassa et al. 2010). It consists of sensory, motor, and autonomic nervous function measurements. Body weight was also recorded. All evaluated signs and symptoms and their assessment are described in Table S1. The parameters were assessed 2 and 24 h after the poisoning. The study was blinded, so the observer did not know the design of the experiment.

Data collected using the FOB included scored and continuous values. Successive statistical tests were used to evaluate constant data: confidence interval for delta, the Barlett test for equality of variances, the William analyses, and the test for distribution functions (Kassa et al. 2017). The chi-squared test of homogeneity, the concordance–discordance test, and the Kruskal–Wallis test assessed the scored values. All tests were performed by SPSS Statistics version 26. The differences were considered significant if *p* ≤ 0.05.

#### Cholinesterase activity in blood and brain

Following FOB, the surviving rats were anesthetized by isoflurane (Werfft, Brno, Czech Republic) vapor at 24.5 h after intoxication and euthanized. The blood was collected from the heart by transcardial puncture using heparinized tubes (Scanlab Systems, Prague, Czech Republic). Subsequently, the brain was swiftly removed and dissected on an ice-cooled plastic pad. The brainstem and cerebellum were removed, and enzyme activity was measured in the forebrain and a small cranial portion of the midbrain. The brain samples were kept at − 80 °C until the final analysis.

Freshly collected whole blood was haemolysed using 20 mM TRIS buffer (pH 7.6, *V*_blood_/*V*_TRIS_ = 1/20). The ChE activity was measured spectrophotometrically by Helios Alpha (Thermo Fisher Scientific) at 436 nm according to the modified Ellman’s method with ATChI as substrate (Ellman et al. 1961). Each concentration of the reactivator was assayed in triplicate.

Brain samples were homogenized in 20 mM TRIS buffer (pH 7.6; *w*_brain_/*w*_TRIS_ = 1/10) using Ultra-Turrax T 18 Digital homogenizer (Thermo Fisher Scientific). The enzyme activity was measured spectrophotometrically by Helios Alpha (Thermo Fisher Scientific) at 412 nm according to the modified Ellman’s method with ATChI as substrate (Ellman et al. 1961). Each concentration of the reactivator was assayed in triplicate.

ChE activity was derived from the absorbance values using the cysteine calibration curve and expressed as μkat/kg or μkat/L (μmol substrate hydrolyzed per kg wet tissue within 1 s or 1 L of blood within 1 s). The blood and brain ChE activity values of the control group were obtained from rats administered physiological saline (B Braun Melsungen AG, Melsungen, Germany) instead of the nerve agent and antidotes (saline control).

The statistical analysis was performed by two-way analysis of variance (ANOVA) with post hoc Dunnett’s multiple comparison test by GraphPad Prism version 9.5.1.

### Molecular modeling

We employed molecular docking and molecular dynamics to calculate the binding poses of our ligands. 3D structures of our ligands were generated by the OpenBabel v. 2.3.2 (O’Boyle et al. 2011), optimized using Avogadro version 1.2.0 with the generalized amber force field (GAFF) (Hanwell et al. 2012), and finally converted into the pdbqt-format by OpenBabel version 2.3.2. The *Hss*AChE structure was obtained from the RCSB database (PDB ID: 5HF9, Crystal structure of *Hss*AChE in complex with paraoxon and HI6, resolution 2.2 Å). The model of A-234-inhibition was created by modifications and prepared for docking using the DockPrep function of Chimera version 1.14 (Pettersen et al. 2004) and MGLTools version 1.5.4 (Morris et al. 2009). Subsequently, the energy minimization of the model was performed using the Gromacs software version 2020.4 (Lindahl et al. 2020). The model was successfully validated by the Verify 3D web tool (https://saves.mbi.ucla.edu/) (Bowie et al. 1991; Lüthy et al. 1992).

The R.E.D. Server (Vanquelef et al. 2011) combined with the Gaussian 16 package revision B.01 (Frisch et al. 2016) was used to calculate the parameters of the serine adducts model with A-234, GA, GB, GD, VX, or paraoxon (POX) ab initio method HF/6-31G*. After that, the Amber14SB force field was manually adjusted and supplemented with new parameters, and the given field was used in molecular dynamics. The Antechamber software version 19.0 was used to parametrize the reactivator models (Wang et al. 2006). The second generation GAFF2 (Trott and Olson 2010) provided the parameters.

The docking calculations were performed using Vina version 1.1.2 (Trott and Olson 2010) as semi-flexible with a flexible ligand and rigid receptor. Docking poses of each reactivator appropriately oriented in the receptor cavity were used as the starting position for molecular dynamic simulation. Gromacs version 2020.4 (Lindahl et al. 2020) carried out MD simulation. The receptor–ligand complex was solvated in the periodic water box using the TIP3P model (Mark and Nilsson 2001). The system was neutralized by adding Cl^−^ and Na^+^ ions to a concentration of 10 nM. The system energy was minimalized and equilibrated in a 100-ps isothermal-isochoric NVT and then a 100-ps isothermal-isobaric NPT phase. Then, a 50-ns MD simulation was run at 300 K. Results were analyzed by the Visual Molecular Dynamics software version 1.9.3.

## Results

### Hydrolysis of A-234

**Fig. 2:**
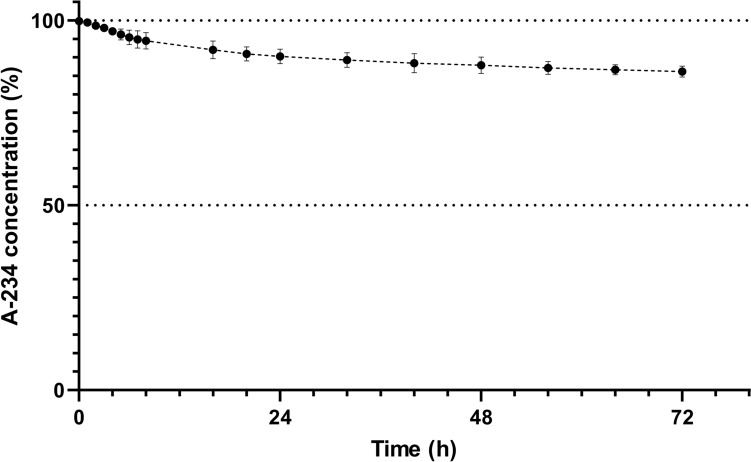
Decrease in A-234 concentration during spontaneous hydrolysis in phosphate buffer pH 7 (mean ± SD, *n* = 3). The experiments were performed in triplicate

The GC-FID was used to determine a kinetic curve of spontaneous hydrolysis of A-234 at 100 µg mL^−1^ in phosphate buffer (pH 7.0) at 25 °C (Fig. 2). To address overall stability (during the first 72 h), the residual concentration of the original amount of spiked analyte was 86.2%. The initial reaction rate (1st h) was 0.027 µM min^−1^. Then the reaction rate decreased to 0.0043 µM min^−1^ (6.3 fold) at the end of the measurement. The reaction rate constant was 9.5 × 10^–5^ min^−1^ (in the 1st h), then increased and remained practically unchanged at 12.2 ± 0.7 × 10^–5^ min^−1^ (2–8 h interval) and then gradually decreased to 3.4 × 10^–5^ min^−1^ at 72 h.

### Biochemical assays

#### Cholinesterase inhibitory assay

The transiently transfected Expi293 cells produced active *Hss*AChE and *Hss*BChE within 7 days, the enzymes were subsequently purified and used for inhibitory and reactivation assays. The inhibitory potency (expressed as IC_50_ ± SEM values) for A-234, GB, and VX against *Hss*AChE and *Hss*BChE are displayed in Table 1. A-234 IC_50_ in *Hss*AChE was 0.101 ± 0.003 µM, which points to the inhibitory efficacy 3.7fold lower than VX but 4.1fold higher than GB. A-234 was the most potent inhibitor of *Hss*BChE (0.036 ± 0.002), with the potency being 17.0 and 29.9 fold higher than GB and VX, respectively.

#### Determination of inhibition rate constants (***k***_***i***_)

The *k*_i_ values for the *Hss*AChE inhibition by A-234, GB, and VX are summarized in Table 2. The most potent OP was A-234 with *k*_*i*_ of 4.3 × 10^8^ M^−1^ min^−1^. The constants for GB and VX were 11.7- and 2.3-fold lower, respectively.

**Table 2:** Inhibitory rate constants of A-234, GB, and VX toward *Hss*AChE (mean ± SEM)

Compound	*k*_*i*_ (M^−1^ min^−1^)
A-234	4.30 ± 0.07 × 10^8^
GB	0.37 ± 0.01 × 10^8^
VX	1.87 ± 0.17 × 10^8^

#### Cholinesterase reactivation assay

**Fig. 3:**
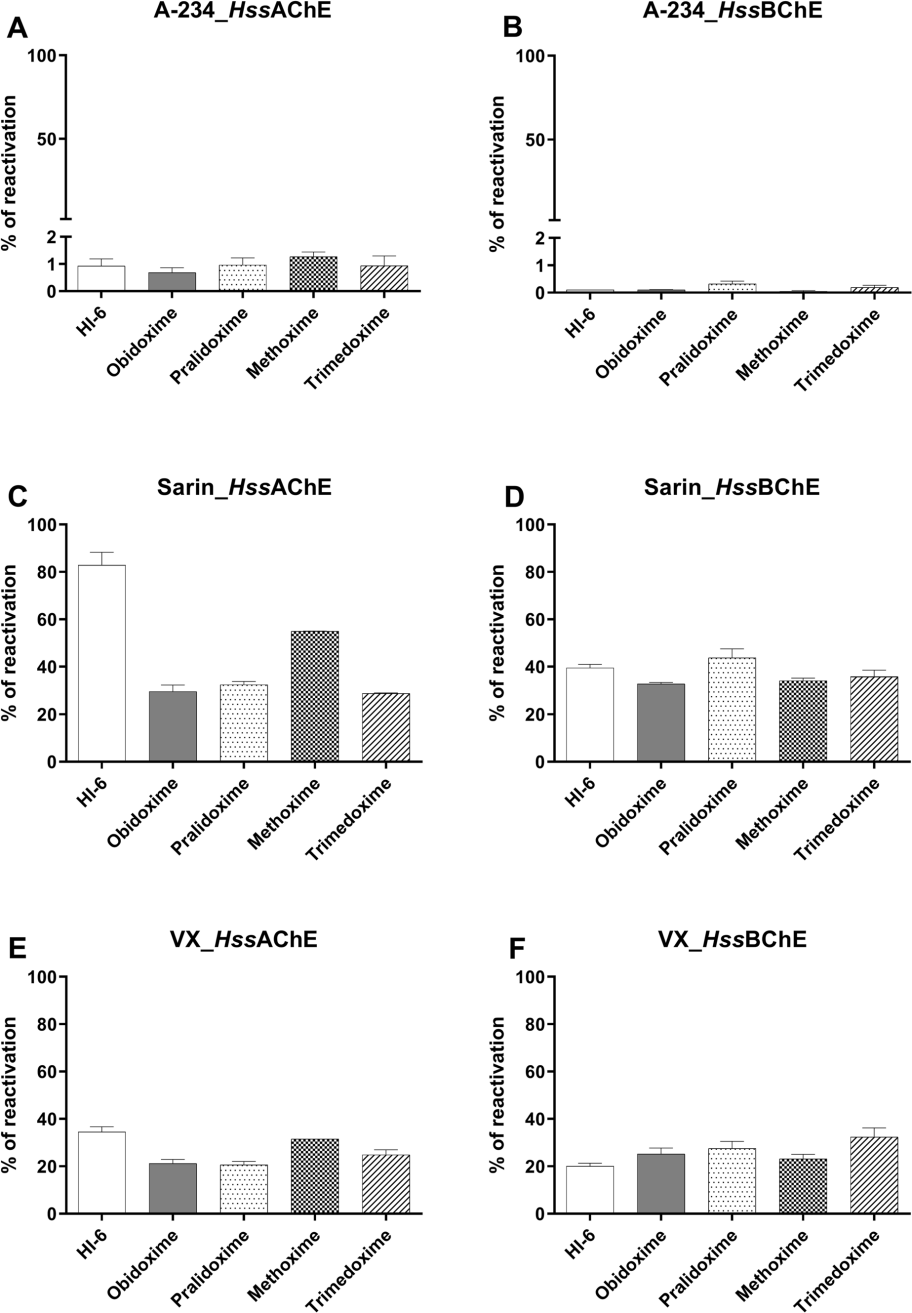
The potency of oximes to reactivate *Hss*AChE and *Hss*BChE (% of reactivation in 10 min) inhibited by A-234, GB, and VX (mean ± SEM, *n* = 3) by HI-6, obidoxime, pralidoxime, methoxime and trimedoxime (100 µM). The experiments were performed in triplicate

The standard oximes possessed very weak reactivation ability at 100 µM toward A-234-inhibited recombinant *Hss*AChE and *Hss*BChE after 10 min. The reactivation ranged 0.68–1.27% for *Hss*AChE (methoxime > pralidoxime > trimedoxime > HI-6 > obidoxime; Fig. 3A) and 0.04–0.32% for *Hss*BChE (pralidoxime > trimedoxime > obidoxime > HI-6 > methoxime; Fig. 3B).

As expected, all oximes showed much higher efficacy against GB and VX. Reactivation of GB-inhibited *Hss*AChE ranged 28.9–82.9% (HI-6 > methoxime > pralidoxime > obidoxime > trimedoxime; Fig. 3C) and 32.9–43.8% for inhibited *Hss*BChE (pralidoxime > HI-6 > trimedoxime > methoxime > obidoxime; Fig. 3D). Reactivation of VX-inhibited *Hss*AChE varied from 20.6 to 34.5% (HI-6 > methoxime > trimedoxime > obidoxime > pralidoxime; Fig. 3E) and 20.0–32.4% for inhibited *Hss*BChE (trimedoxime > pralidoxime > obidoxime > methoxime > HI-6; Fig. 3F).

#### Reactivation kinetics assessment

HI-6 and methoxime were selected for subsequent reactivation kinetics measurements due to the highest reactivation efficacy towards GB- and VX-inhibited *Hss*AChE. Both tested reactivators showed no efficacy in reactivating *Hss*AChE inhibited by A-234 (see the supplementary material Fig. S3 and S4). The most efficient reactivator of GB- and VX-inhibited enzyme was HI-6. HI-6 had the highest affinity (the lowest *K*_*D*_ values) to both OPs-inhibited enzyme. Regarding methoxime, its reactivity (*k*r) was similar to that of HI-6. However, due to the lower affinity for GB- and VX-inhibited *Hss*AChE, its reactivation rate constants (*k*_*r2*_) were lower than for HI-6 (Table 3).

**Table 3:** Reactivation constants for HI-6- and methoxime-induced reactivation of GB- and VX-inhibited *Hss*AChE

	A-234		GB		VX	
	HI-6	Methoxime	HI-6	Methoxime	HI-6	Methoxime
*k*_r_ (min^−1^)	NC	NC	1.68	1.22	1.49	1.64
*K*_D_ (µM)	NC	NC	18.90	74.74	7.20	113.6
*k*_r2_ (mM^−1^ min^−1^)	NC	NC	88.68	16.35	206.64	14.46
*R*^2^	NC	NC	0.988	0.993	0.995	0.998

The maximal first-order reactivation rate constant (*k*_r_), the dissociation constant of the enzyme-oxime complex (*K*_D_), and the overall second-order reactivation rate constant (*k*_r2_) were determined from at least three experiments at 37 °C. *R*^2^ is the correlation coefficient

*NC* not calculated due to low reactivation

### In vivo experiments

#### Evaluation of selected countermeasures' therapeutic efficacy

A broad spectrum of muscarinic (salivation, lacrimation, and others) and nicotinic (tonic–clonic convulsions) symptoms was observed in all poisoned rats within a few min after administering A-234, regardless of the antidote. At lower doses of A-234, atropine partially suppressed and postponed muscarinic symptoms, whereas diazepam tended to reduce nicotinic signs. The therapeutic effectiveness of atropine alone was generally lower than in combination with diazepam and selected oximes. Atropine with diazepam provided a protective ratio of 2.61. Obidoxime in the triple combination did not increase survival. Methoxime in a triple combination reduced the A-234 acute lethality 3.46-fold, while HI-6 showed to be the most effective, with a protective ratio of 5.61. Nonetheless, we observed no significant differences between the diazepam and all the oxime groups, including the HI-6 group (Table 4). Despite this, we included methoxime and HI-6 in the follow-up behavioral study because both oximes displayed higher antidotal efficacy than obidoxime.

**Table 4:** Overview of medicines/antidotes to reduce the A-234 lethality in rats

Poisoning/Treatment	*i.m.* LD_50_ (95% CI) (μg/kg)	Protective ratio versus the untreated group	Protective ratio versus the atropine group
A-234	13.5 (12.0–15.6)	–	–
Atropine (10 mg/kg)	25.0 (18.2–31.8)*	1.85	–
Diazepam (2.5 mg/kg) + atropine	35.2 (30.6–41.8)*^#^	2.61	1.41
Obidoxime (10.5 mg/kg) + atropine and diazepam	35.0 (28.3–42.7)*^#^	2.59	1.40
Methoxime (22.1 mg/kg) + atropine and diazepam	40.4 (31.9–47.4)*^#^	2.99	1.62
HI-6 (39 mg/kg) + atropine and diazepam	75.8 (37.0–157.7)*^#!^	5.61	3.03

*Significantly different from the untreated group based on relative median potency (RMP)

^#^Significantly different from the atropine group based on RMP

^!^Chi-square analysis did not detect parallelism between the HI-6 group and the untreated or atropine group. Therefore, RMP analysis may not provide accurate results

#### Functional observatory battery

In this behavioral study, the A-234 was administered in a dose corresponding to 90% of previously established *i.m.* LD_50_ (12.2 µg/kg). The therapy consisted only of atropine and oximes. Diazepam was omitted due to its effects on the central nervous system, which could alter the results of FOB.

During the 24 h, 3 of 8 unprotected rats died. The poisoning significantly affected 14 of 41 parameters 2 h after the intoxication. It reduced the number of rearings, length of landing foot splay, pupil size and response to light, and respiration while increasing repetitive movements of mouth and jaws, tremors, ataxia score, forelimb grip strength, catch difficulty score, and touch response. Simultaneously, we observed significantly uncoordinated air-righting reflex from the vertical position, impaired gait score due to ataxia, and fur abnormalities (piloerection and fur dishevelment). At 24 h after the poisoning, only 8 of 41 parameters were changed compared with control animals. The intoxication reduced the animal posture score, although it fell into the physiological category. It increased rearing activity, hyperkinesis, clonic movements, and the total disability score. We also observed persisting reduced landing foot splay and slow touch response, while we found no response to click.

All the chosen treatment strategies fully protected the animals from death, although numerous symptoms of intoxication were observed. Mydriasis at 2 h indicates atropinization in all three groups. Compared with intoxicated animals, each therapy approach improved only 3 parameters while aggravating others in this time interval. Better outcomes were seen at 24 h, with atropinization alone or atropine-HI-6 treatment having the best results. These two groups improved 5 and 4 parameters compared to the unprotected group, respectively. In contrast, hindlimb grip strength remained reduced in the atropine group, and only fur abnormalities were aggravated in atropine-HI-6-treated rats. The observed symptoms defining sensory, motor, and autonomic nervous functions are summarized in the supplementary material (Table S2, S3, and S4).

#### Cholinesterase activity in blood and brain

**Fig. 4:**
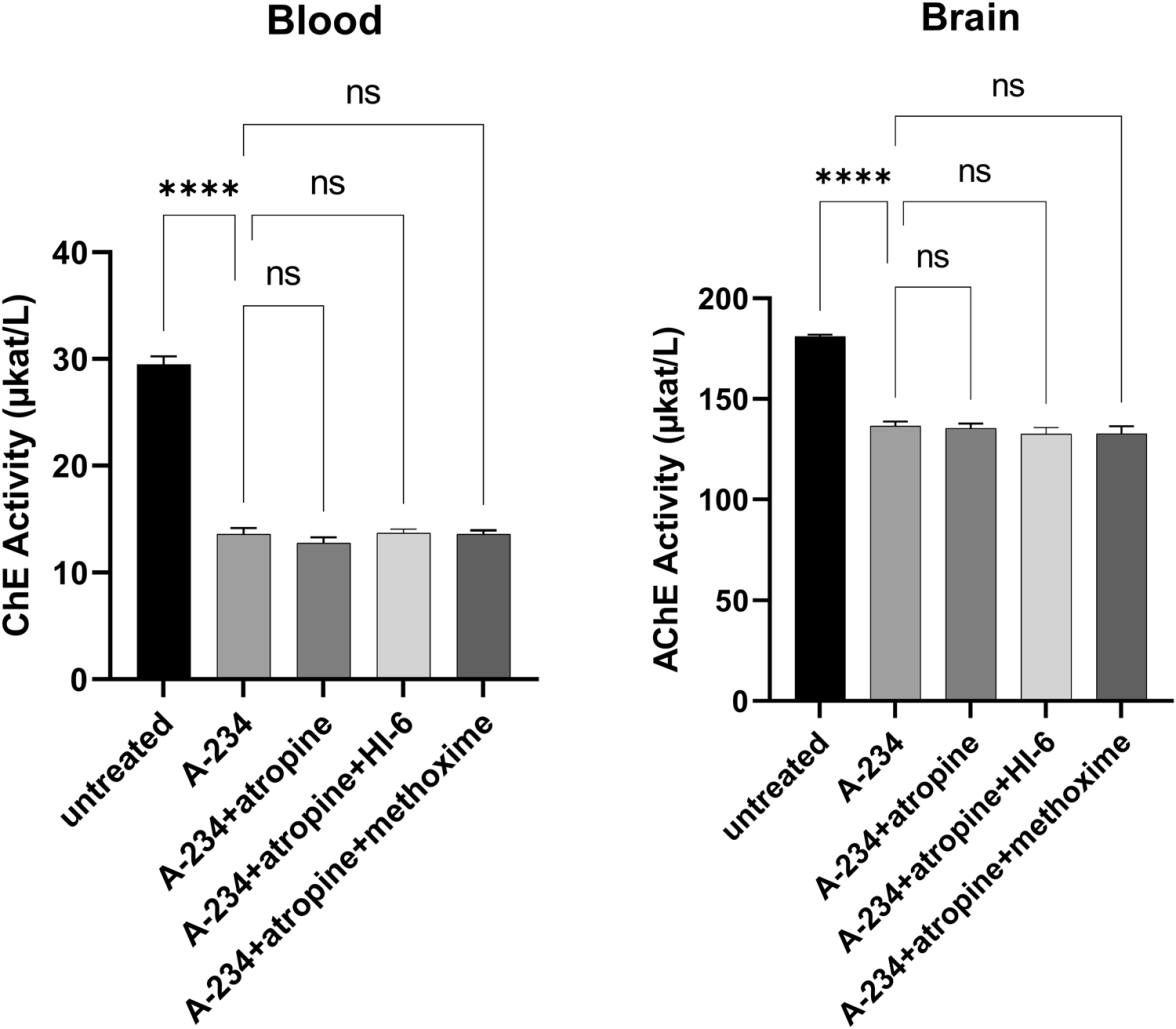
AChE reactivation with tested oximes in blood and brain in rats intoxicated by A-234. The results are expressed as mean ± SEM (*n* = 8, except for A-234, where *n* = 5). Dosing corresponds to Table 4

ChE activities in blood and the brain were used as bioindicators of A-234 toxic effects 24.5 h after the poisoning. The reactivation effectiveness was tested in rats treated with atropine alone or combined with HI-6 or methoxime. In A-234 exposed animals, both oximes showed no reactivation in the blood and brain (Fig. 4).

### Molecular modeling

Pralidoxime showed the shortest distance between the oxygen of the reactivator oxime group and A-234 phosphorus. For all oximes, the distance ranged from 3.21 to 7.75 Å (pralidoxime < obidoxime < methoxime < trimedoxime < HI-6, Table 5). The 50-ns molecular dynamics simulation results showing the interaction course are also in supplementary Fig. S5–S9. The interactions of all five oxime reactivators with inhibited *Hss*AChE are additionally depicted in supplementary material in Fig. S10–S14.

**Table 5:** The molecular modeling result overview includes the minimum oxime oxygen-A-234 phosphorus (O–P) distance, time of reaching the minimum, interaction energy defined as the sum of short-range Lennard–Jones and Coulombic interaction energies, and partial charge of the oxime oxygen

Ligand	Minimum O–P Distance in Å (time of reaching the minimum)	Interaction energy (kJ mol^−1^)	Partial charge
HI-6	7.75 Å (at 17.7 ns)	− 271.8	− 0.403
obidoxime	3.37 Å (at 25.3 ns)	− 341.1	− 0.310
pralidoxime	3.21 Å (at 49.7 ns)	− 75.2	− 0.409
methoxime	3.44 Å (at 43.7 ns)	− 296.4	− 0.397
Trimedoxime	5.83 Å (at 47.4 ns)	− 175.5	− 0.331

Obidoxime displayed the highest interaction energy, while pralidoxime had the lowest. The interaction energy ranged from − 75.2 to − 341.1 kJ mol^−1^ (obidoxime > methoxime > HI-6 > trimedoxime > pralidoxime, Table 5).

All five oximes possessed low partial charge on the oxygen of the oxime group, ranging from − 0.310 to − 0.397 (Table 5). By contrast, the phosphorus partial charge in A-234-inhibited serine showed the lowest values compared with other selected OPs (Table 6).

**Table 6:** Phosphorus partial charge in OP-inhibited serine calculated by the R.E.D (Vanquelef et al. 2011)

Nerve agent	Phosphorus partial charge
A-234-serine	0.522
GA-serine	0.849
GB-serine	0.959
GD-serine	0.946
POX-serine	1.116
VX-serine	0.958

Server combined with the Gaussian 16 package revision B.01 (Frisch et al. 2016) with A-234, GA, GB, GD, POX, or VX ab initio method HF/6-31G*

## Discussion

Although the incidents that made A-234 famous happened in 2018 (Lewis 2018; Carlsen 2019), information and publically available experimental preclinical data about the so-called Novichok agents remain scarce. Understanding the properties and effects of A-series agents can not only dispel myths surrounding the compounds but also support the development and optimization of medical countermeasures.

We started our experiments by monitoring the spontaneous hydrolysis of A-234 in neutral conditions to determine the stability of the compound for the following experiments. The results showed a slow degradation process kinetics. After 72 h, the residual concentration of the original amount of spiked analyte was 86.2%. Interestingly, the reaction rate displayed a triphasic course. The reaction rate was increased during the first phase. Harvey et al. determined the rate of hydrolysis to be 0.0032 µM min^−1^ at 10 min (25 °C in 50 mM Bis–tris-propane, pH 7.2), suggesting an even slower initial reaction speed (Harvey et al. 2020). In the following phase, the reaction rate stabilized for at least 6 h and then gradually decreased. Using GraphPad Prism and data from the 16–72 h interval, it is possible to calculate the half-life of approximately 14 days. Such a result corresponds with Mirzayanov, who estimated the hydrolysis half-life of A-234 to be 10–30 days at pH 6.5–7.4 (Mirzaynov 2008). Nevertheless, due to changing reaction kinetics, we can only firmly claim that the half-life exceeds 72 h. Other studies assessing the degradation of A-234 in a neutral environment (pH 7–7.2) performed the measurements only for several minutes up to 1 h, concluding that the compound is stable (Harvey et al. 2020; de Koning et al. 2022; Jung et al. 2023). There is one exception. Lee et al. studied the fragmentation pathway of A-234 under different pH conditions. They found that A-234 was completely degraded in a neutral aqueous solution (pH 7.2) after two months. However, they could not draw any reaction kinetics because they solely evaluated the initial and the 2-month interval endpoints (Lee et al. 2021). They also observed that the main fragmentation product was ethylhydrogen (1-(diethylamino)ethylidene)phosphoramidate, while diethylacetamidine and ethylhydrogen phosphofluoridate were minor products. The observed multiple degradation pathways could explain the changing hydrolysis rate.

Following the stability tests, we performed in vitro experiments on the inhibitory activity of OPs against human ChEs. A-234 proved to be a potent inhibitor of both enzymes. Its inhibition efficacy towards *Hss*AChE was lower than VX but higher than GB. By contrast, A-234 showed the highest inhibitory potential towards *Hss*BChE. Compared with *Hss*AChE*, Hss*BChE has a broader dimension of the active-site gorge (Masson et al. 2009), making it more accessible for bulkier structures like A-234. However, the reason for the different inhibitory trends remains unknown. It could be related to the micro-environment of the enzyme gorge as it can significantly determine the selectivity of inhibitors for ChEs (Saxena et al. 1999). In any case, high *Hss*BChE sensitivity to A-234 emphasizes the feasibility of the enzyme in the therapy for scavenging A-234 stoichiometrically (Nachon et al. 2013). Early administration of fresh frozen plasma or recombinant enzyme may prevent the distribution or redistribution of A-234 in the body. Steindl et al. administered fresh frozen plasma in the only documented A-234 case report. The patient received six plasma units on day 6, which led to a pronounced increase in BChE activity with no subsequent decline excluding the presence of an unbound inhibitory agent in blood. On the other hand, it did not cause any clinical improvement (Steindl et al. 2021).

Another in vitro parameter that quantifies the inhibitory potency of specific agents and may provide an initial estimate of the toxic potential is determining the *k*_i_ with *HssA*ChE (Worek et al. 2020). In this regard, A-234 seems to be more potent inhibitor than GB and VX. Notably, values measured for GB and VX also correspond to results published by Worek et al. (Worek et al. 2002, 2004, 2020). In vitro determination of the bimolecular inhibition rate constant *k*_*i*_ with recombinant *Hss*AChE allows quantification of the inhibitory potencies of A-234, GB, and VX and may provide an initial estimate of the toxic potential of these agents. The nerve agent A-234, with a *k*_*i*_ 4.30 ± 0.07 × 10^8^ M^−1^ min^−1^, was more potent agent than GB and VX. For GB and VX it is 0.367 ± 0.007 and 1.87 ± 0.17 × 10^8^ M^−1^ min^−1^, respectively. Similar trends were observed in the study Worek et al. 2020 (Worek et al. 2020).

As a next step, we tested the reactivation efficacy of five marketed oxime reactivators (HI-6, obidoxime, pralidoxime, methoxime, and trimedoxime). These reactivators are more or less successfully used against OP poisoning (Dhuguru et al. 2022). None of the reactivators appeared to be able to efficiently reactivate AChE inhibited by all OPs regardless of their structure (Worek et al. 2007). Our results show that all five compounds can counteract VX and GB poisoning but fail against A-234. None of them reached at least 10% reactivation of inhibited AChE, which is the recommended reactivation potency affecting the prognosis of an intoxicated organism (Kuca et al. 2009). The following kinetic study confirmed the results of the reactivation screening for HI-6 and methoxime. HI-6 showed very low reactivation (2–3% after 50 min) of A-234-inhibited *Hss*AChE even at the highest concentrations (0.025 mM). Methoxime showed no reactivation potency of A-234 inhibited *Hss*AChE. Thus, kinetic constants (*k*_r_, *K*_D,_ and *k*_r2_) could not be calculated. By contrast, HI-6 and methoxime effectively reactivated GB- and VX-inhibited *Hss*AChE, with HI-6 having a higher affinity and reactivation rate than methoxime. These results are consistent with the work of Worek et al. 2004, 2010, despite the fact that the real values differ between the studies, which can be ascribed to different source of the enzyme.

The following experiments aimed to determine A-234 toxicity and assess different therapeutical approaches in rats. Notably, the only available scientific knowledge on the toxicity of A-series compounds so far is based on computational modeling (Jeong and Choi 2019; De Farias 2019; Bhakhoa et al. 2019; Carlsen 2019). However, such studies can sometimes provide conflicting data. For instance, Carlsen et al. used the Toxicity Estimation Software Tool (T.E.S.T.) to calculate the oral LD_50_ values of rats, which was then extrapolated to human toxicity following the guide for dose conversion between animals and humans based on the body surface area. According to their results, A-234 should be 7.1 less toxic than VX (Carlsen 2019). On the other hand, De Farias employed semi-empirical (PM6) molecular modeling and argued for higher A-234 toxicity than VX due to fewer conformers implied in forming possible linkages to AChE (De Farias 2019). Regarding symptoms, we could only find small pieces of information in medical management guidelines published by the US government. The guideline indicated bronchoconstriction and seizures as a prominent feature of A-series agent toxicity in animals with the risk of severe metabolic acidosis documented by markedly elevated serum lactate (U.S. Government 2019). Our results showed that the toxicity of A-234 after i.m. exposure is high and very close to that of VX (13.5 µg/kg for A-234 vs. 10 µg/kg for VX, data for VX not shown). Indeed, lethal doses of A-234 induced a wide range of muscarinic and nicotinic symptoms in rats, including seizures at supralethal doses.

The therapy of OP poisoning usually consists of atropine (or other anticholinergics) to reverse the muscarinic effects of acetylcholine, diazepam (or other drugs inhibiting the central nervous system) for neuroprotection, and oxime to restore AChE physiologic function (Pulkrabkova et al. 2022). Although in vitro experiments indicated oxime inefficacy, we included them in therapeutic regimes as the settings of in vitro experiments exclude mechanisms that may play an essential role in vivo, such as a direct reaction of oximes with unbound OP (Becker et al. 2007) or other non-cholinergic effects, like a direct modulation of cholinergic receptors (van Helden et al. 1996; Soukup et al. 2011b, 2013). To assess the protective ratio, we selected three oximes based on availability in the Czech Republic. Obidoxime is the only oxime approved for treating OP poisonings in the civilian sector (Kuca et al. 2009), while the Czech army is equipped with HI-6 and methoxime (Bajgar 2012). The results confirmed that atropine and diazepam are effective drugs regardless of the nerve agent nature. By contrast, oximes did not provide significant benefits, although methoxime and HI-6 substantially increased the protective index compared to atropine and diazepam combination.

The second in vivo model (FOB) helped to explain this observation at least partially. Given the same degree of ChE inhibition in all intoxicated groups, the oximes did not reactivate rat AChE or significantly interact with an unbound agent to affect its toxicokinetics. Due to the short oxime half-life in the circulation (Lundy et al. 2011), their effects can be expected relatively shortly after administration. Compared to animals treated with atropine alone, both HI-6 and methoxime positively affected only tremors in the 2 h interval. Tremors can be associated with the overstimulation of nicotinic receptors (Liu et al. 2015), while oximes have been found to modulate nicotinic transmission at several levels (Soukup et al. 2011a, 2013). Although the selected oximes showed a different impact on the protective indices, the finding correlated well with the administered oxime dose as they were not administered equimolarly but at doses corresponding to 5% of their respective LD_50_ (Kuca et al. 2013).

The clinical use of oximes against A-234 thus remains questionable. According to the U.S. medical management guidelines, British clinicians treating victims of A-234 poisoning noticed that high doses of pralidoxime helped maintain the blood pressure and renal perfusion and, therefore, may provide some benefit (U.S. Government 2019). On the other hand, oximes affect liver functions and may induce hepatotoxicity, especially at high doses (Pejchal et al. 2008; Horn et al. 2023). Indeed, Steindl et al. treated an A-234 victim with obidoxime. After one day, they discontinued the infusion because elevated transaminases and γ-glutamyl transferase were observed, and there was no sign of reactivation or improvement in neuromuscular functions (Steindl et al. 2021).

Finally, we utilized molecular dynamics modeling and identified three parameters to find explanations for oxime reactivation ineffectiveness against A-234. The first parameter represents the minimal distance between oxime oxygen and A-234 phosphorus reached during simulations. Although the optimum range for this parameter to indicate reactivation has not yet been determined, the oxygen should be able to approach the phosphorus atom at the van der Waals contact distance (approx. 3.3 Å). Using molecular dynamics modeling, Gerlits et al. demonstrated that RS-170B, an imidazole-based aldoxime, could bring its oxime oxygen as close as 3.4 Å to the VX phosphorus in inhibited AChE, thus producing a near-attack configuration (Gerlits et al. 2019). By contrast, Gorecki et al. considered the distance of approximately 5 Å still favorable for nucleophilic attack (Gorecki et al. 2021). From this perspective, HI-6 and trimedoxime reactivation moieties appear unable to reach their target. On the other hand, obidoxime, pralidoxime, and methoxime exceeded or came close to the van der Waals contact distance (3.21–3.44 Å). The second parameter, interaction energy, reflects the stability of the oxime-A-234-AChE complex, which contributes to the probability of nucleophilic attack. Gorecki et al. perceived 210–250 kJ/mol as a favorable range (Gorecki et al. 2021). Thus, the interaction energy for obidoxime and methoxime seems optimal but low for pralidoxime. However, even sufficient proximity and adequate interaction energy do not imply that reactivation will occur. Quantum mechanics complementary to molecular study may provide better insight (da Silva et al. 2020), but our study has not utilized this approach. To circumvent this deficit, we calculated the partial charge of the oxime oxygen. The calculations showed that tested oximes are relatively weak nucleophiles. Considering the low A-234 phosphorus partial charge (lower than the other OPs) and that A-234 can form stabilizing hydrophobic or electrostatic interactions with the choline-binding site (Radić 2021), a stronger nucleophile seems necessary for effective binding and subsequent reactivation. The low A-234 phosphorus partial charge implicates the reactivation failure of all oximes synthesized so far.

## Conclusion

Information about A-series compounds, including A-234, is modest. Some represent newspaper interviews with scientists who came across these compounds during their careers. Interestingly, in 1997, a Washington Times article mentioned that “these new agents are as toxic as VX, as resistant to treatment as soman, and more difficult to detect and easier to manufacture than VX “(Gertz 1997). Our research has confirmed that the first two statements are factual in the case of A-234. Its high stability, toxicity, and resistance to reactivators available for the treatment are critical for managing poisoned victims. From a therapeutic point of view, anticholinergics supplemented with a neuroprotective agent are the mainstay of treating A-234 poisoning. However, the possible reactivation of inhibited ChEs remains unresolved and will pose a challenge for further research. Possibly, entirely different structures may be necessary to restore their physiological functions.

### Supplementary Information

Below is the link to the electronic supplementary material.

Supplementary file1 (TIF 107 KB)

Supplementary file2 (TIF 55 KB)

Supplementary file3 (TIF 1559 KB)

Supplementary file4 (TIF 1537 KB)

Supplementary file5 (TIF 486 KB)

Supplementary file6 (TIF 488 KB)

Supplementary file7 (TIF 384 KB)

Supplementary file8 (TIF 555 KB)

Supplementary file9 (TIF 512 KB)

Supplementary file10 (TIF 209 KB)

Supplementary file11 (TIF 299 KB)

Supplementary file12 (TIF 197 KB)

Supplementary file13 (TIF 332 KB)

Supplementary file14 (TIF 297 KB)

Supplementary file15 (DOCX 29 KB)

Supplementary file16 (DOCX 35 KB)

Supplementary file17 (DOCX 28 KB)

Supplementary file18 (DOCX 30 KB)

Supplementary file19 (DOCX 1316 KB)

## Data Availability

Data will be available upon request from authors.
