# Supplementary figures and images for: A-series agent A-234: initial in vitro and in vivo characterization
Source: Arch Toxicol. 2024 Mar 6;98(4):1135–49. doi: 10.1007/s00204-024-03689-3 (PMC10944400; doi:10.1007/s00204-024-03689-3)

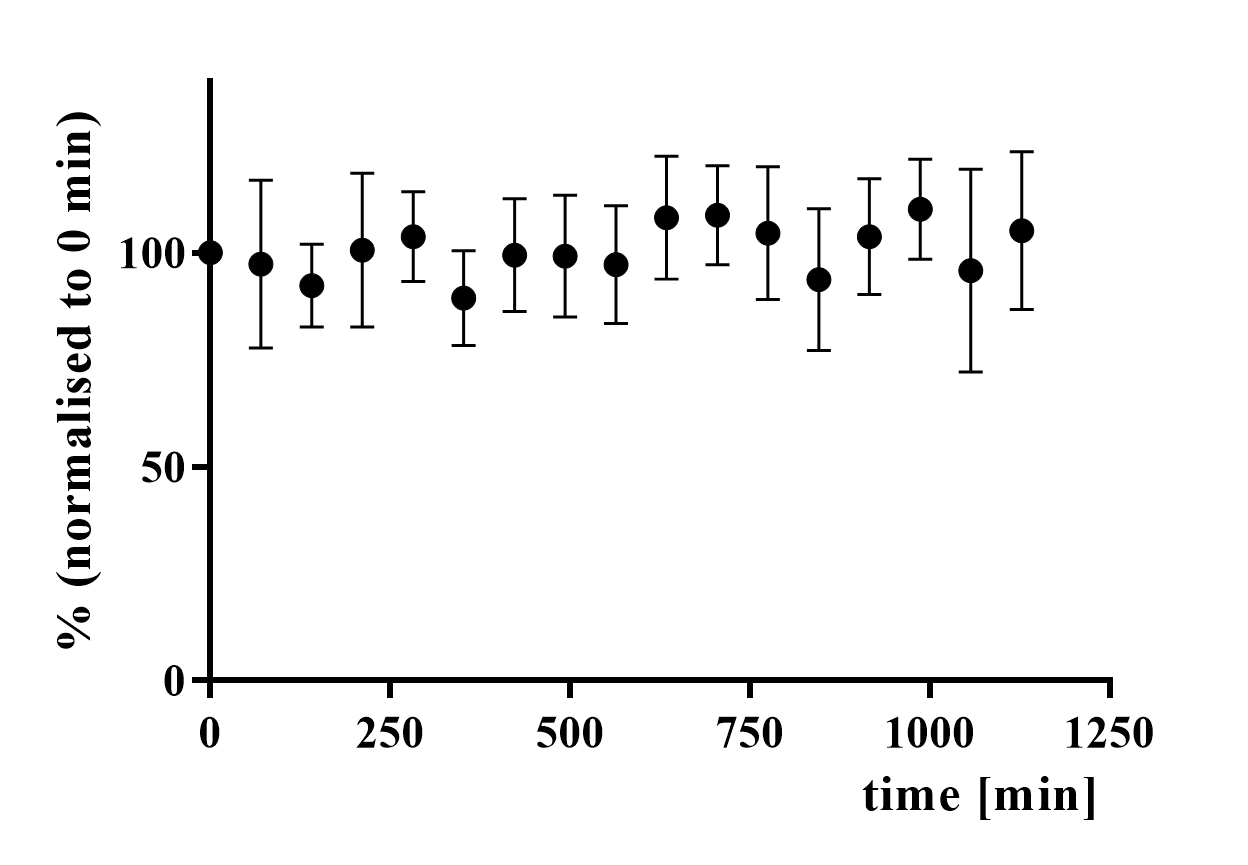

Supplement: Supplementary file 1 — Supplementary file1 (TIF 107 KB) [file 204_2024_3689_MOESM1_ESM.tif]

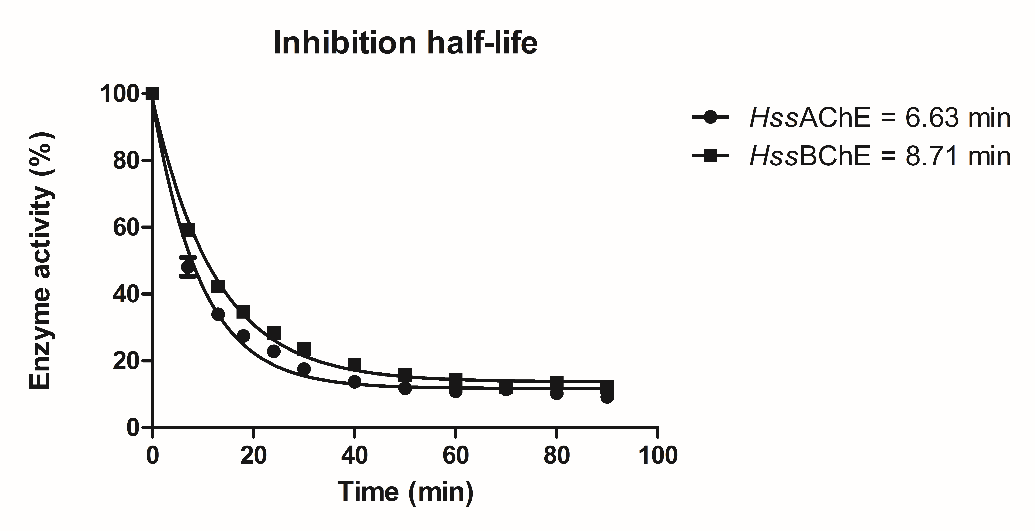

Supplement: Supplementary file 2 — Supplementary file2 (TIF 55 KB) [file 204_2024_3689_MOESM2_ESM.tif]

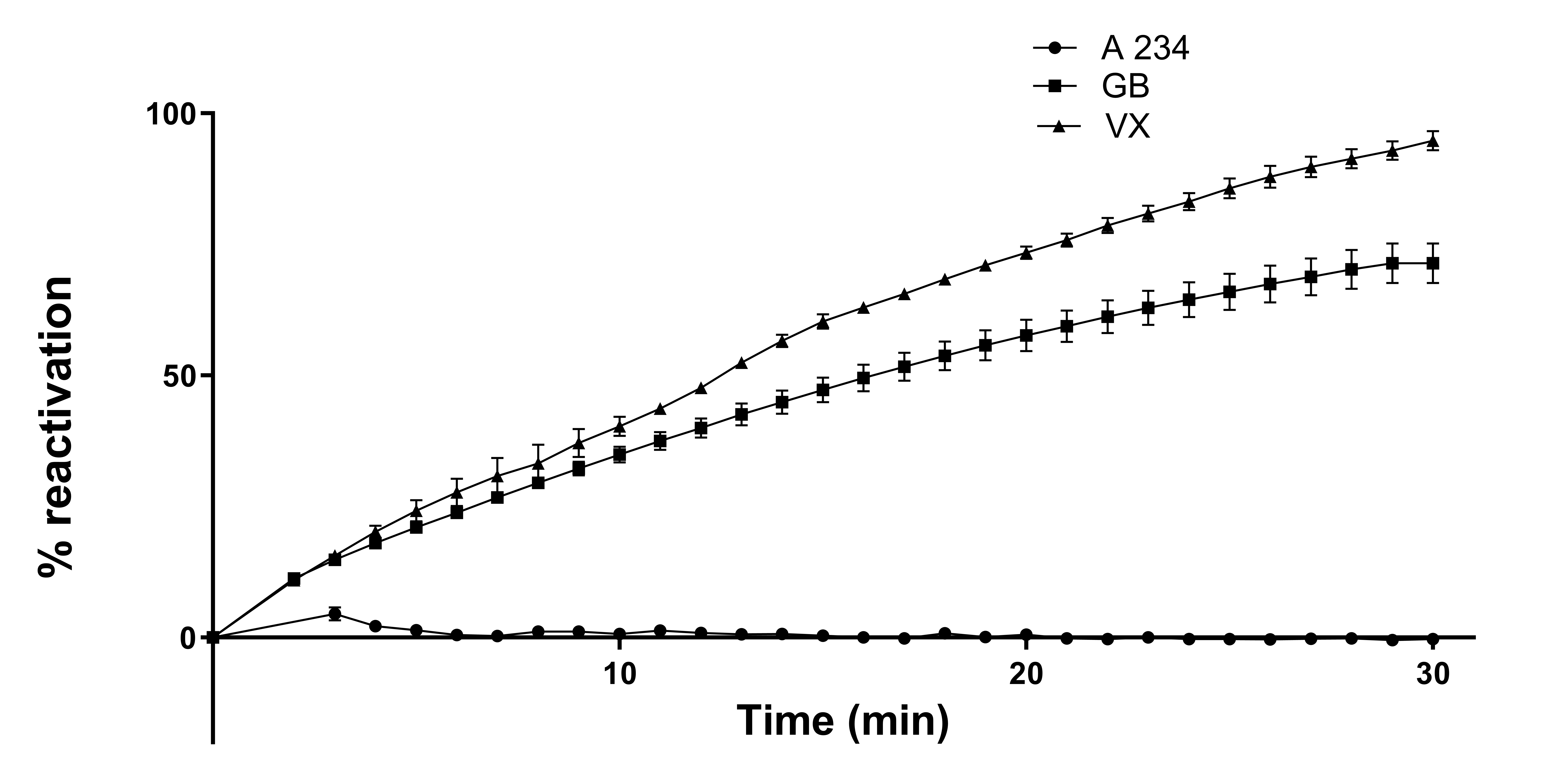

Supplement: Supplementary file 3 — Supplementary file3 (TIF 1559 KB) [file 204_2024_3689_MOESM3_ESM.tif]

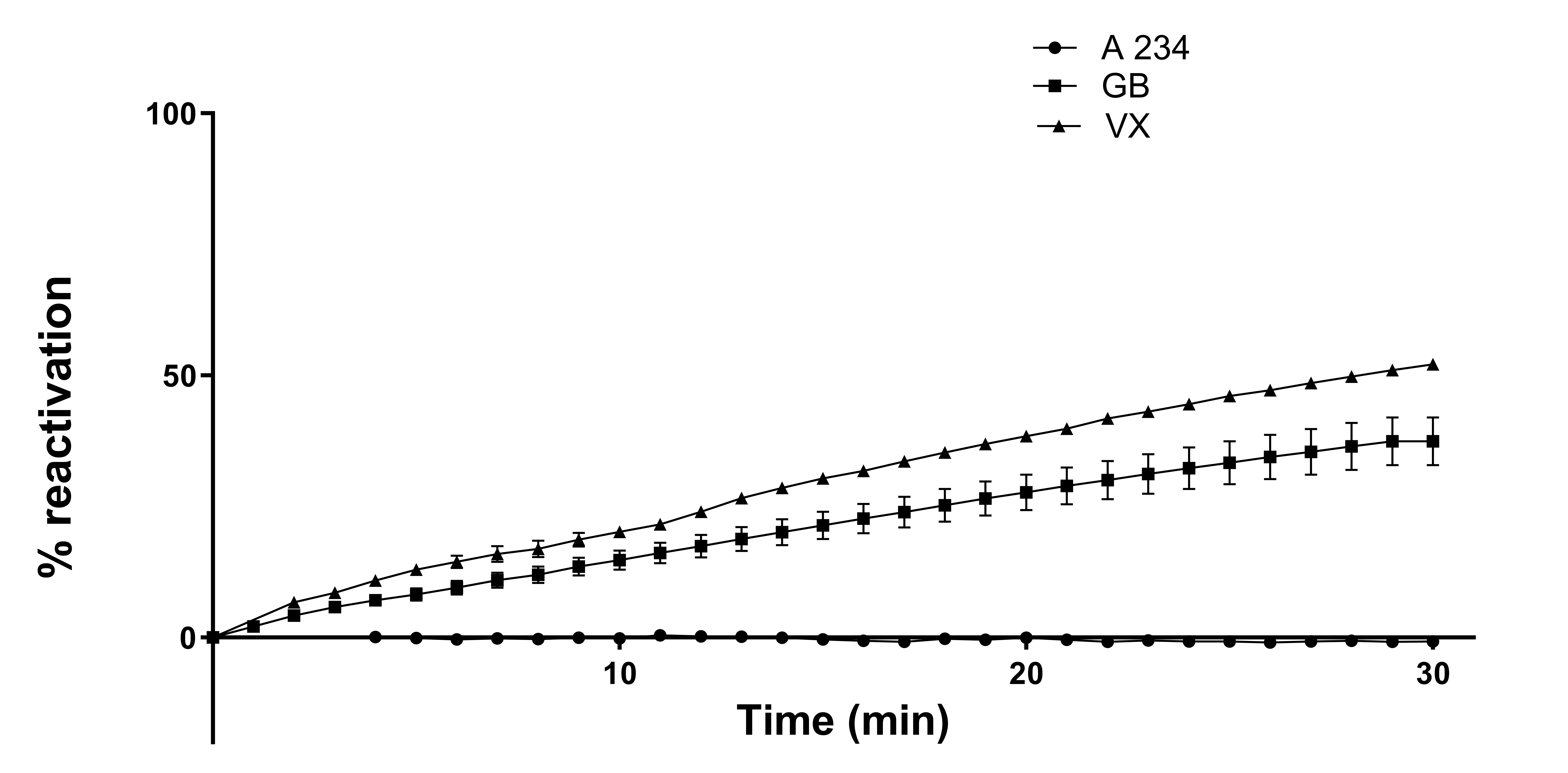

Supplement: Supplementary file 4 — Supplementary file4 (TIF 1537 KB) [file 204_2024_3689_MOESM4_ESM.tif]

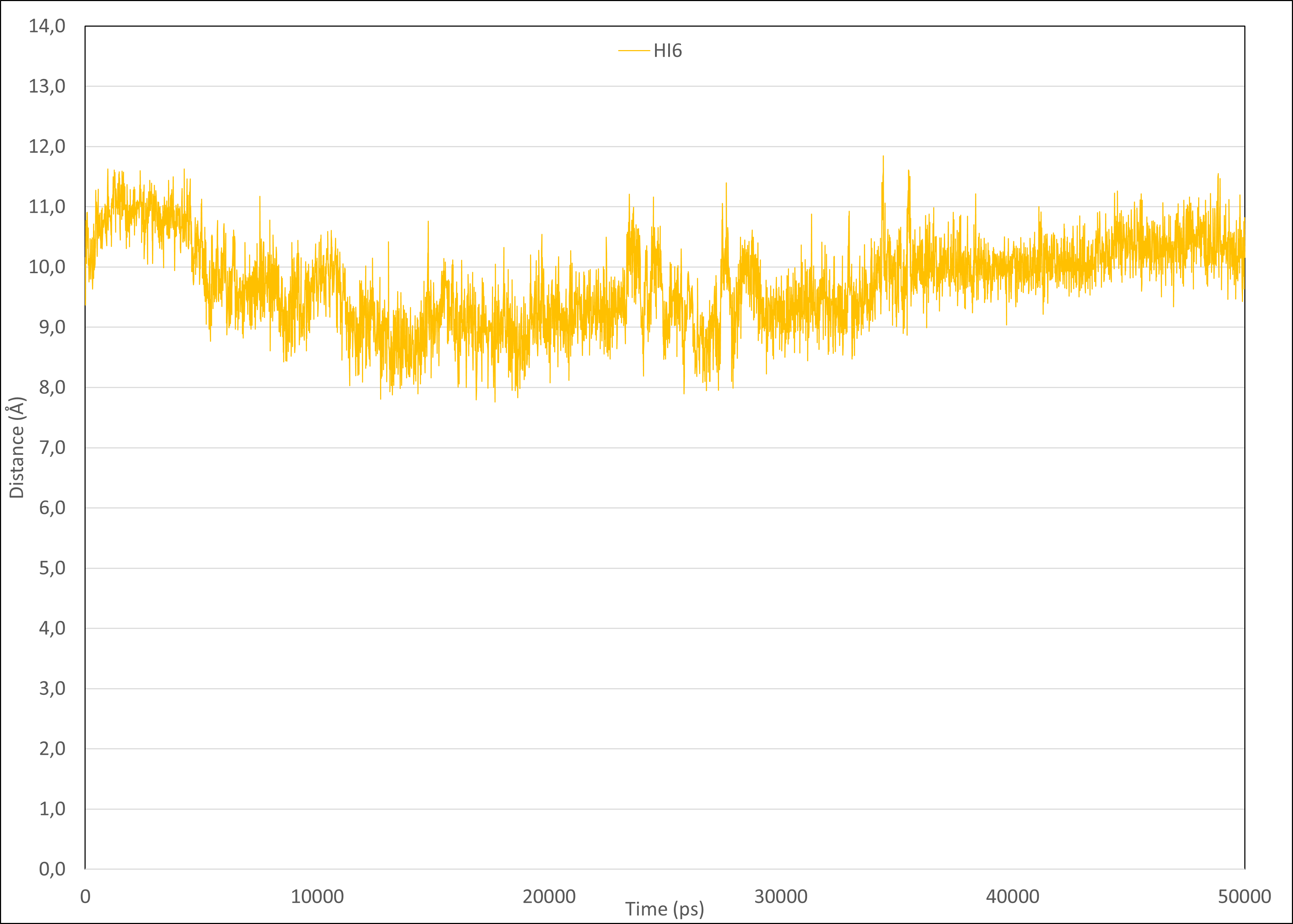

Supplement: Supplementary file 5 — Supplementary file5 (TIF 486 KB) [file 204_2024_3689_MOESM5_ESM.tif]

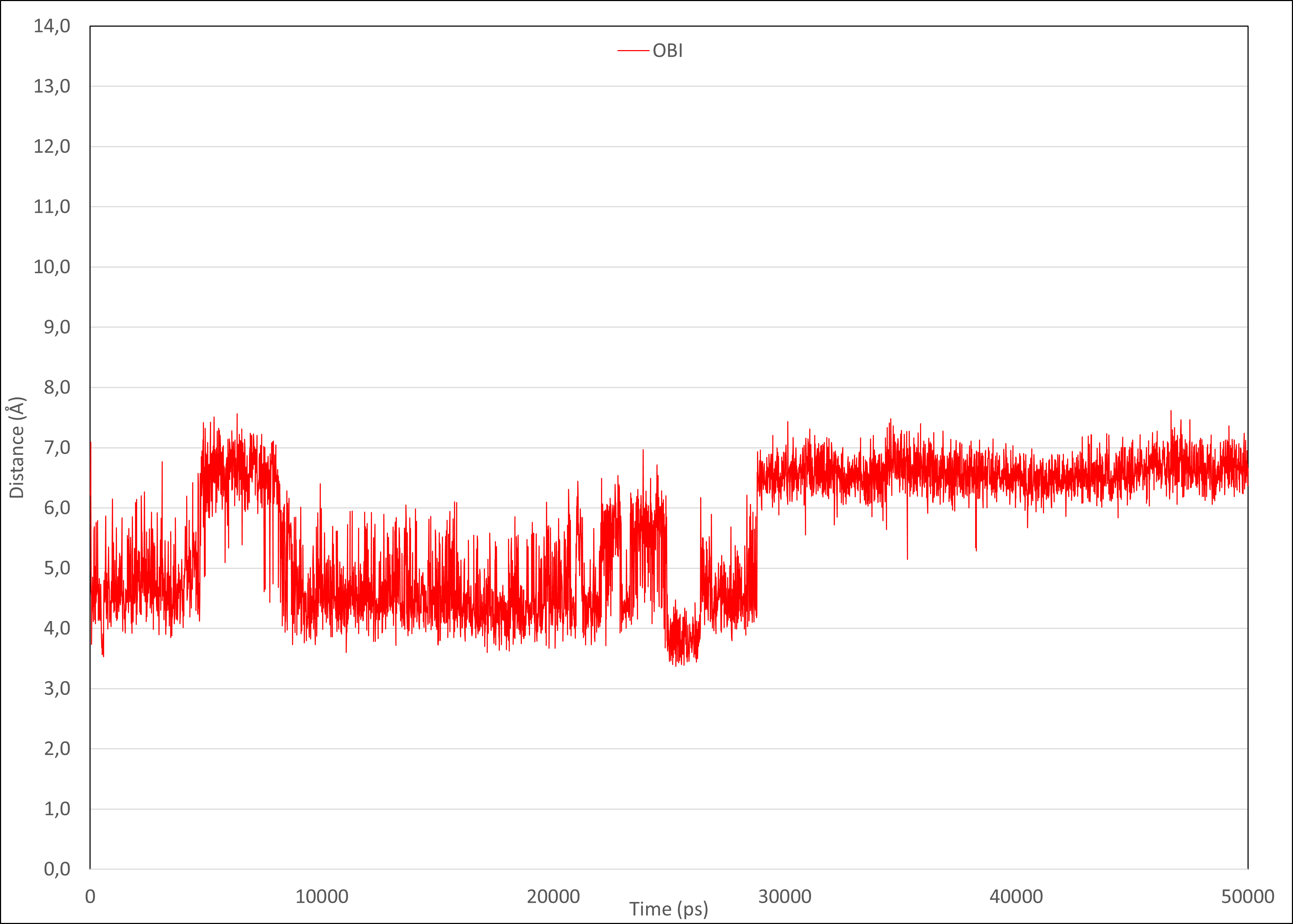

Supplement: Supplementary file 6 — Supplementary file6 (TIF 488 KB) [file 204_2024_3689_MOESM6_ESM.tif]

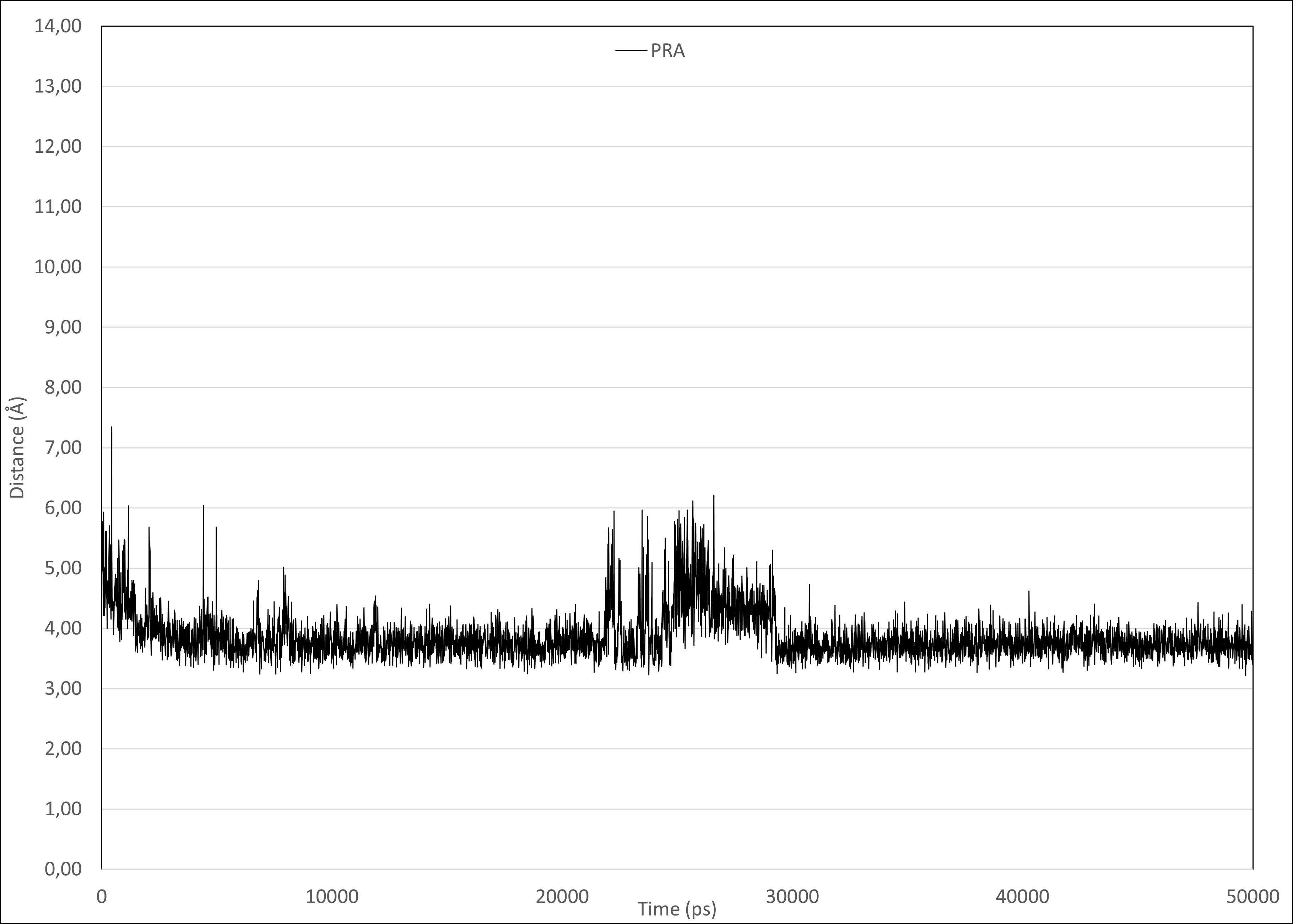

Supplement: Supplementary file 7 — Supplementary file7 (TIF 384 KB) [file 204_2024_3689_MOESM7_ESM.tif]

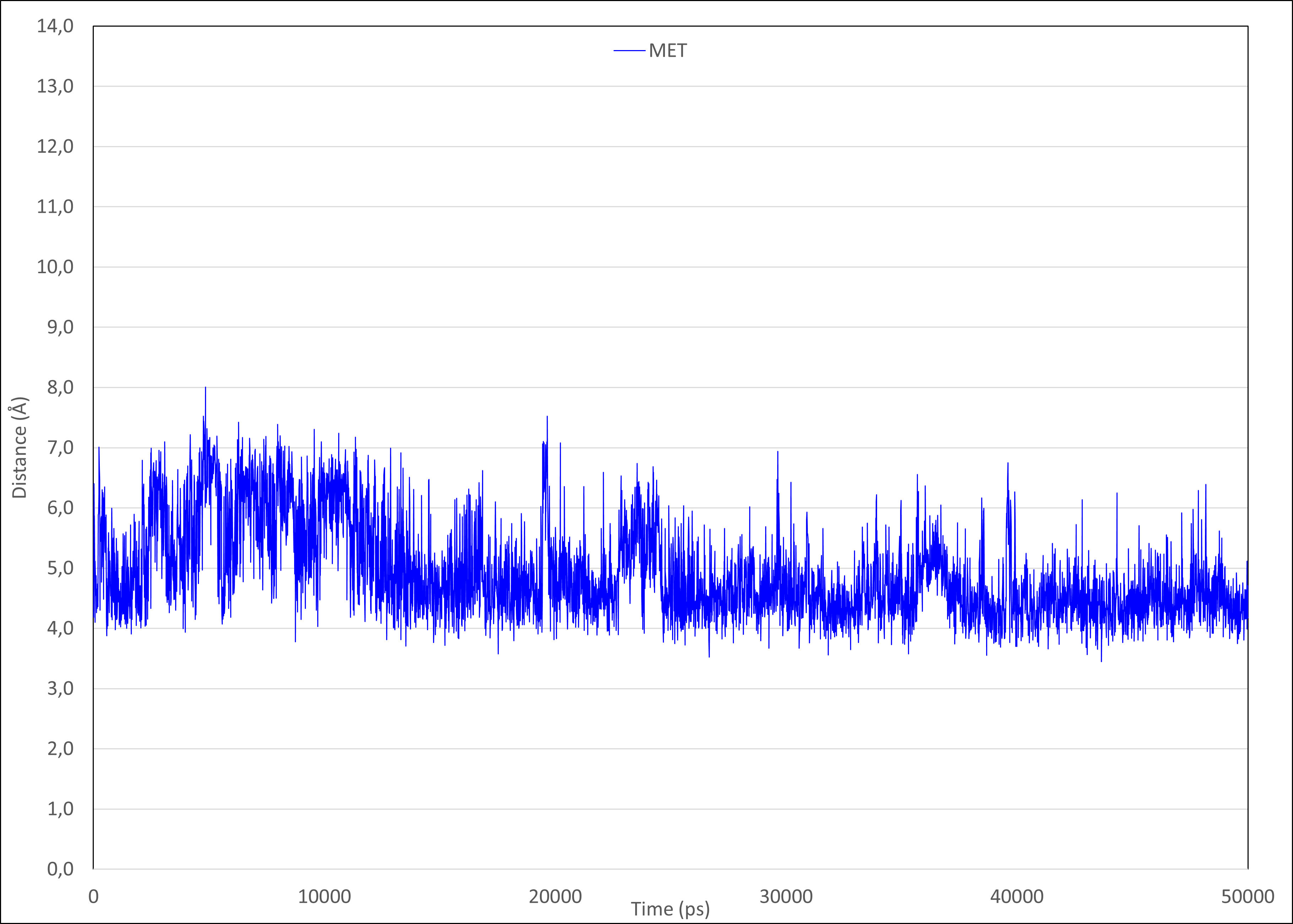

Supplement: Supplementary file 8 — Supplementary file8 (TIF 555 KB) [file 204_2024_3689_MOESM8_ESM.tif]

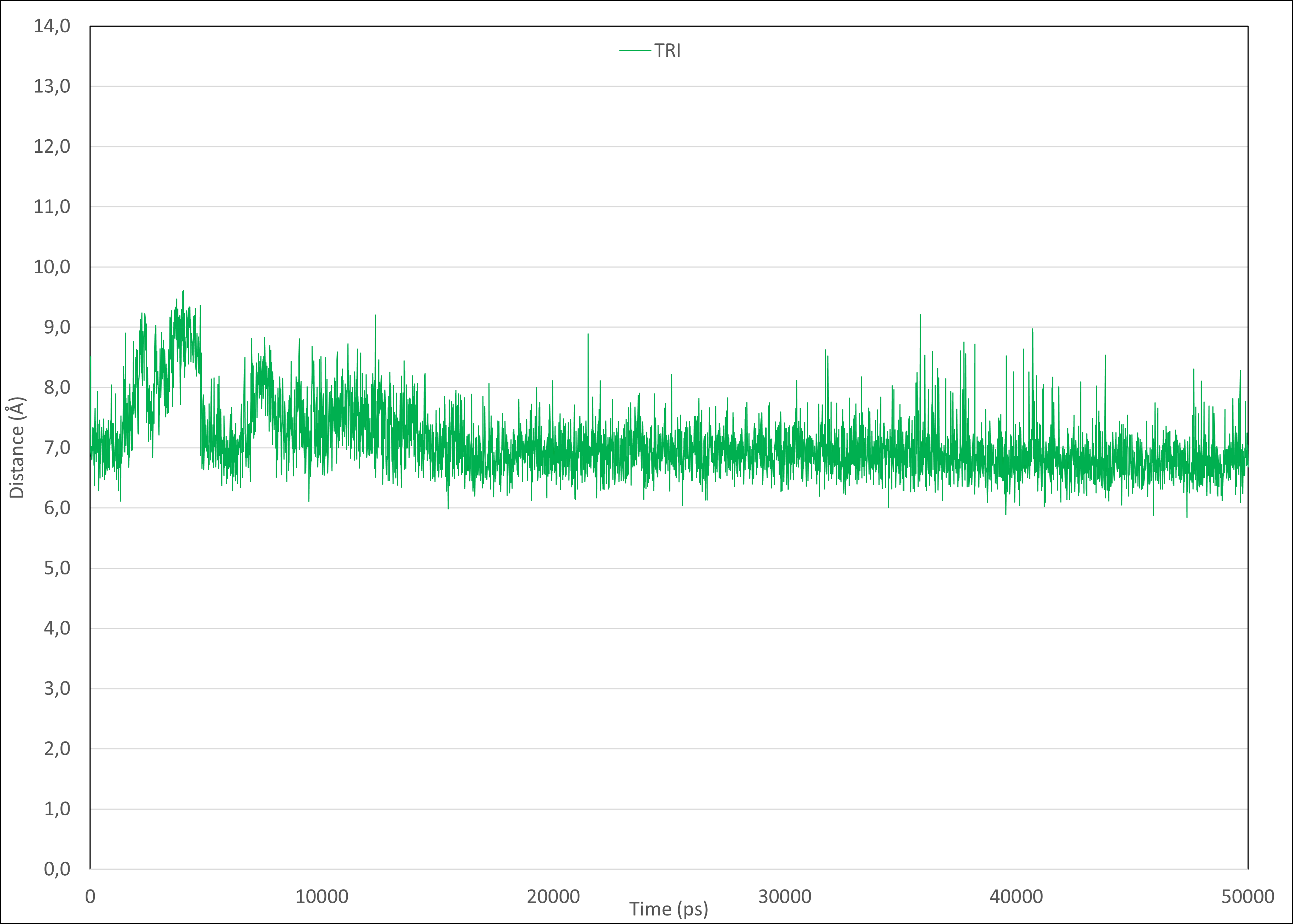

Supplement: Supplementary file 9 — Supplementary file9 (TIF 512 KB) [file 204_2024_3689_MOESM9_ESM.tif]

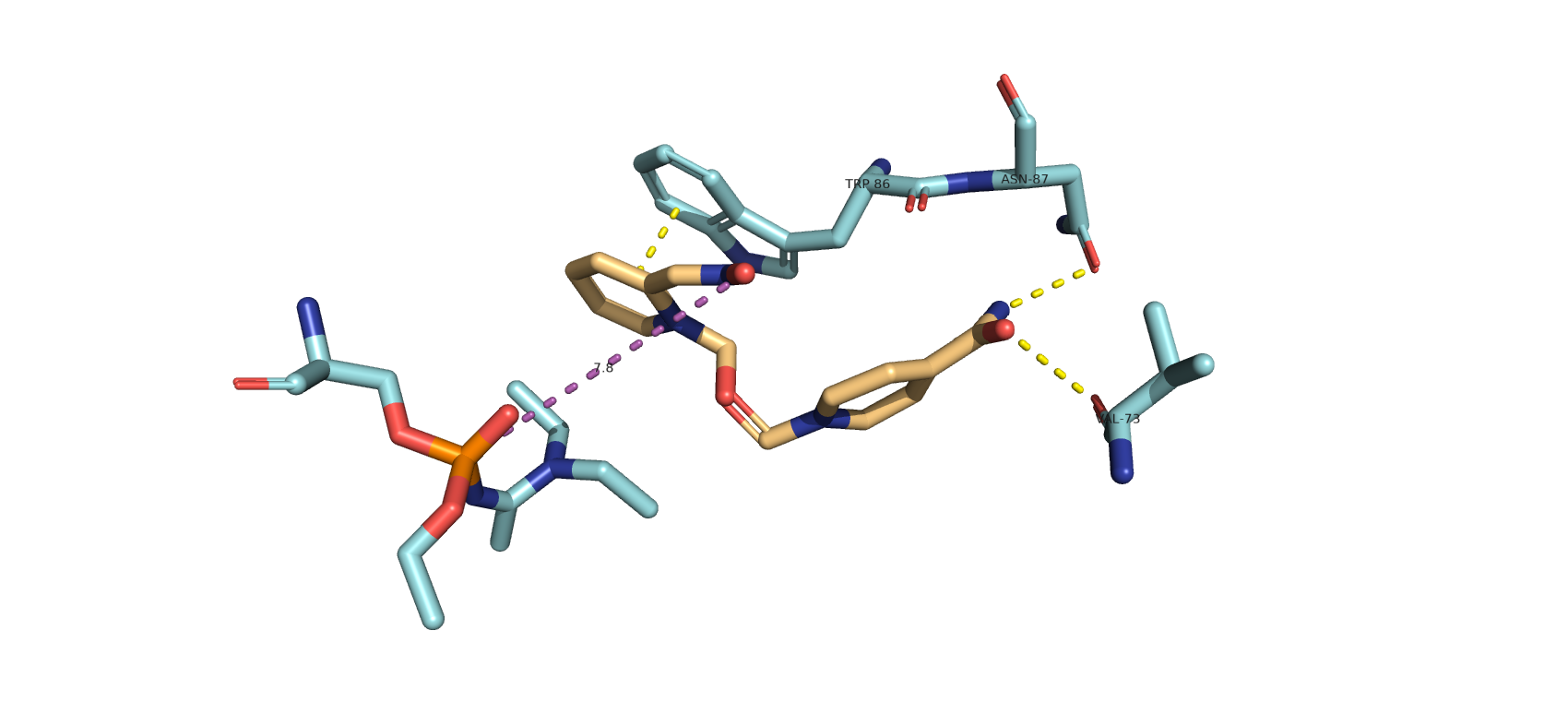

Supplement: Supplementary file 10 — Supplementary file10 (TIF 209 KB) [file 204_2024_3689_MOESM10_ESM.tif]

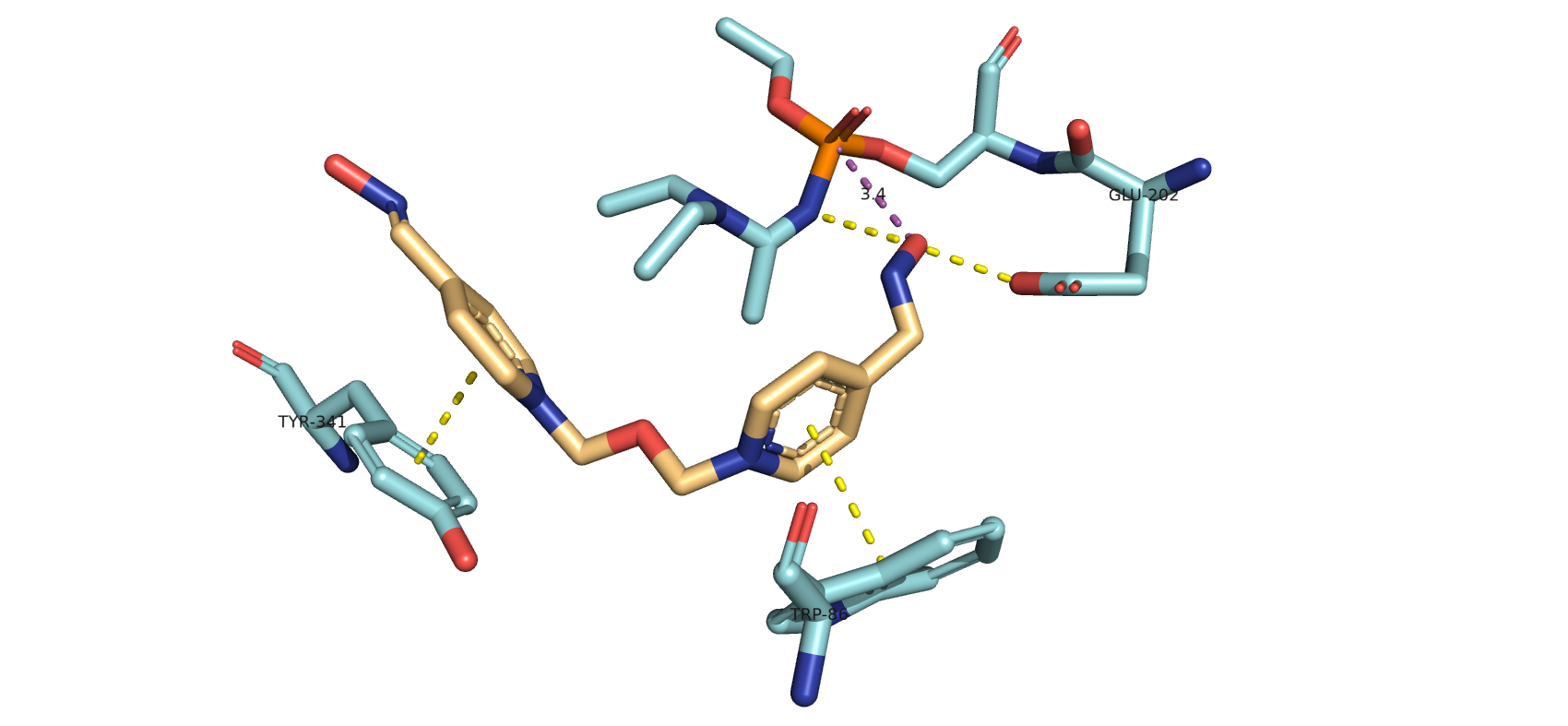

Supplement: Supplementary file 11 — Supplementary file11 (TIF 299 KB) [file 204_2024_3689_MOESM11_ESM.tif]

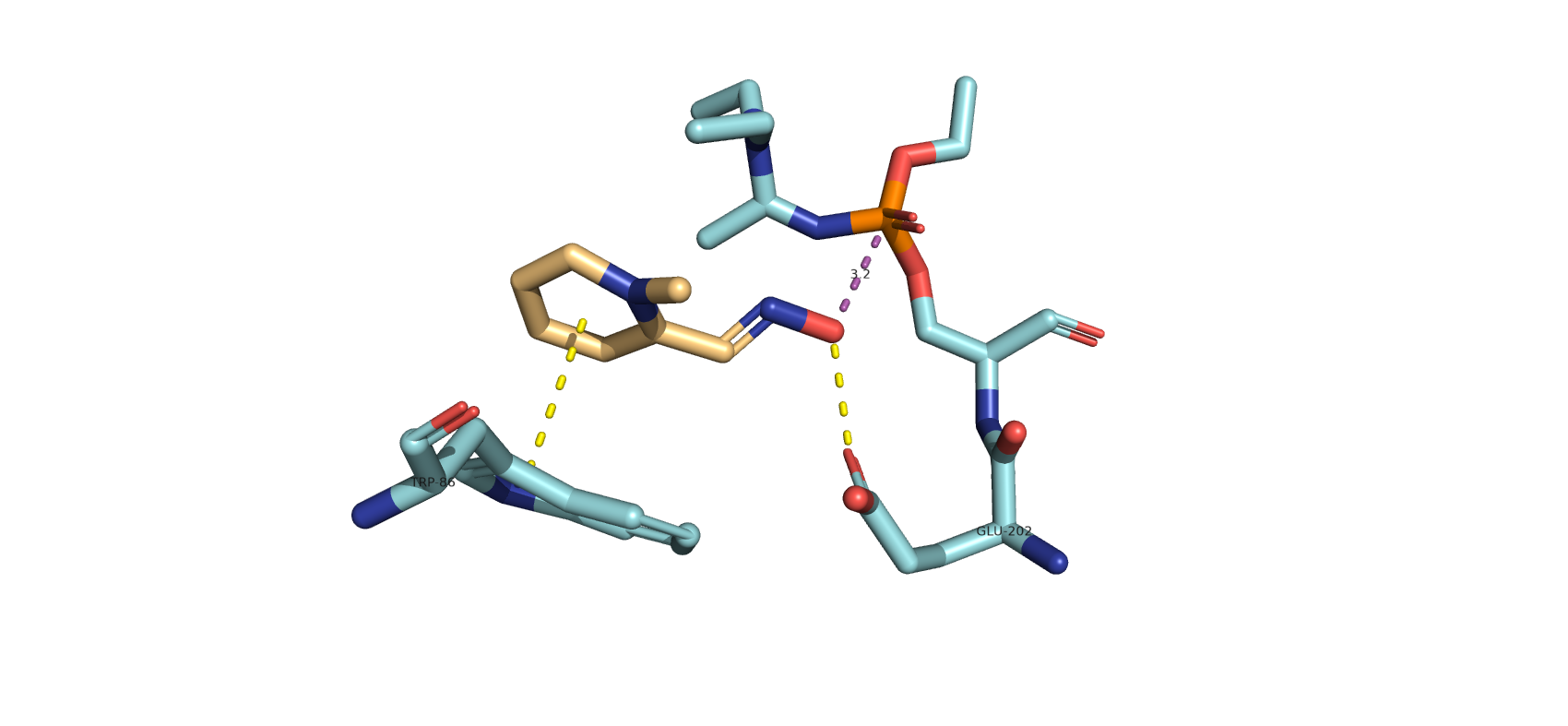

Supplement: Supplementary file 12 — Supplementary file12 (TIF 197 KB) [file 204_2024_3689_MOESM12_ESM.tif]

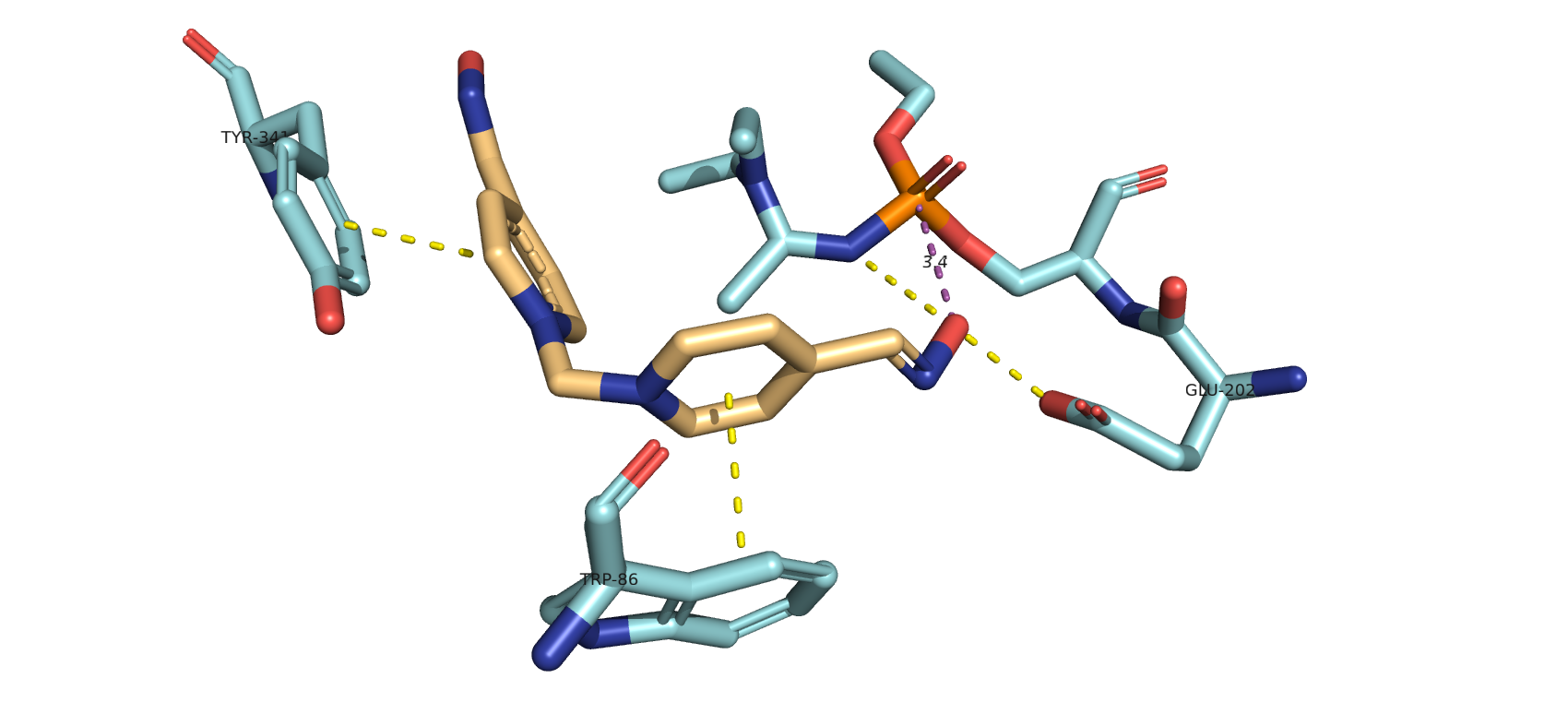

Supplement: Supplementary file 13 — Supplementary file13 (TIF 332 KB) [file 204_2024_3689_MOESM13_ESM.tif]

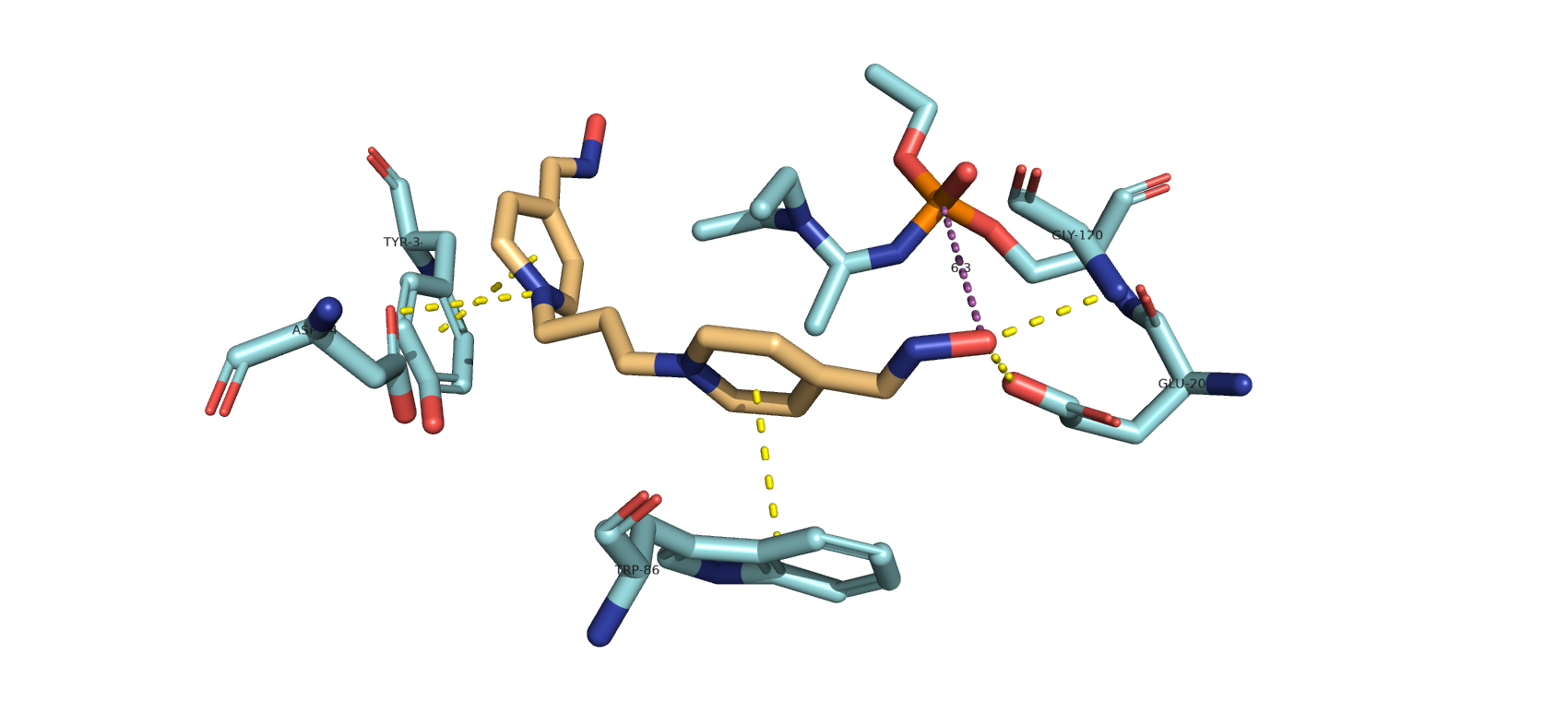

Supplement: Supplementary file 14 — Supplementary file14 (TIF 297 KB) [file 204_2024_3689_MOESM14_ESM.tif]
