# Supplementary material for: A-series agent A-234: initial in vitro and in vivo characterization
Source: Arch Toxicol. 2024 Mar 6;98(4):1135–49. doi: 10.1007/s00204-024-03689-3 (PMC10944400; doi:10.1007/s00204-024-03689-3)
Supplement: Supplementary file 15 — Supplementary file15 (DOCX 29 KB) [file 204_2024_3689_MOESM15_ESM.docx]

**Table S1**. Signs and symptoms assessed in the functional observatory battery and their semi-quantitative scales.

| **Marker** | **Scored values** | | | | | | | | | |
| --- | --- | --- | --- | --- | --- | --- | --- | --- | --- | --- |
|  | **-2** | **-1** | **0** | **1** | **2** | **3** | **4** | **5** | **6** | **7** |
| **posture** |  |  |  | ***sitting or standing*** | ***rearing*** | ***asleep*** | flattened | lying on side | crouched over | head bobbing |
| **muscular tonus** | atonic | hypotonic | ***normal*** | hypertonic | rigidity | fasciculation |  |  |  |  |
| **hyperkinesis** |  |  | ***normal***  ***kinesis*** | repetitive movements of mouth and jaws | non-rhythmic quivers | mild tremors | severe tremors | myoclonic jerks | clonic convulsion |  |
| **tremors** |  |  | ***none*** | after stimulation | mild local | mild overall | medium–influencing movement | strong–impeding movement |  |  |
| **clonic movements** |  |  | ***none*** | twitches | non-rhythmic movement |  |  |  |  |  |
| **tonic movements** |  |  | ***normal*** | contraction of extensors | opistotonus | emprostotonus | explosive jumps | tonic convulsions |  |  |
| **gait** |  |  | ***normal*** | ataxia | overcompensation of hind limb movements | feet point outwards from the body | forelimbs are extended | walks on tiptoes | hunched body | the body is flattened against the surface |
| **ataxia** |  |  | ***none*** | mild | severe |  |  |  |  |  |
| **total disability score** |  |  |  | ***normal*** | slightly impaired | somewhat impaired | totally impaired |  |  |  |
| **mobility score** |  |  |  | ***normal*** | slightly impaired | somewhat impaired | totally impaired |  |  |  |
| **activity** |  |  |  | very low | sporadic | reduced | ***normal*** | enhanced | permanent |  |
| **righting reflex (from vertical or back position)** |  |  |  | ***normal*** | slightly uncoordinated | lands on side | lands on back |  |  |  |
| **catch difficulty** |  |  |  | passive | ***normal*** | defense | flight | escape | aggression |  |
| **ease of handling** |  |  |  | very easy | ***easy*** | moderately difficult | difficult |  |  |  |
| **tension** |  |  | ***none*** | partial (ears) | stupor |  |  |  |  |  |
| **vocalization** |  |  | ***none*** | provoked | spontaneous | excessive |  |  |  |  |
| **stereotypy** |  |  | ***none*** | head weaving | body weaving | grooming | circling | others |  |  |
| **bizarre behavior** |  |  | ***none*** | head | body | self-mutilation | abnormal movements | others |  |  |
| **approach response** |  |  |  | no reaction | ***normal*** | slow reaction | energetic reaction | exaggerated reaction |  |  |
| **touch response** |  |  |  | no reaction | ***normal*** | slow reaction | energetic reaction | exaggerated reaction |  |  |
| **click response** |  |  |  | no reaction | ***normal*** | slow reaction | energetic reaction | exaggerated reaction |  |  |
| **tail-pinch response** |  |  |  | no reaction | ***normal*** | slow reaction | energetic reaction | exaggerated reaction |  |  |
| **lacrimation** |  |  | ***none*** | slight | severe | crusts | colored crusts |  |  |  |
| **lids position** |  |  |  | ***open*** | slightly dropping | half-way dropping | completely shut | ptosis |  |  |
| **endo/exophthalmos** |  | enophthalmos | ***normal*** | exophthalmos |  |  |  |  |  |  |
| **fur abnormalities** |  |  | ***normal*** | colored | disheveled | colored and disheveled | baldness | injury | other changes | piloerection |
| **skin abnormalities** |  |  | ***normal*** | pale | erythema | cyanosis | pigmentation | cold | injury |  |
| **salivation** |  |  | ***none*** | slight | severe |  |  |  |  |  |
| **nose secretion** |  |  | ***none*** | slight | severe | colored |  |  |  |  |
| **pupil size** |  | miosis | ***normal*** | mydriasis |  |  |  |  |  |  |
| **pupil response** |  |  | no reaction | ***normal reaction*** |  |  |  |  |  |  |
| **respiration** | apnea | bradypnea | ***normal*** | tachypnea | dyspnea |  |  |  |  |  |

Signs and symptoms described in Bold italics represent the physiological range.

Symptoms, such as rearing (per min), urination (area of urine drops on absorbent paper), and defecation (number of poops), were directly quantified, the quantification period lasted for 3 min.

Landing foot splay (cm) and body weight (g) were measured by ruler and weight (A&D company, Tokyo, Japan), respectively.

Hindlimb, forelimb grip strength, and strength of all limbs were measured using a strength meter (Electronika, Praha, ČR).
