# Supplementary material for: A-series agent A-234: initial in vitro and in vivo characterization
Source: Arch Toxicol. 2024 Mar 6;98(4):1135–49. doi: 10.1007/s00204-024-03689-3 (PMC10944400; doi:10.1007/s00204-024-03689-3)
Supplement: Supplementary file 16 — Supplementary file16 (DOCX 35 KB) [file 204_2024_3689_MOESM16_ESM.docx]

**Table S2.** Overview of activity and neuro-muscular parameters assessed 2 and 24 h after the A-234 challenge (90% of LD_50_).

|  | 2 hours | | | | | | | | | | | | | | |
| --- | --- | --- | --- | --- | --- | --- | --- | --- | --- | --- | --- | --- | --- | --- | --- |
|  | saline-saline | | | A234-saline | | | A234-atropine | | | A234-atropine-methoxime | | | A234- atropine-HI-6 | | |
|  | modus/mean | SD | modus/mean | | SD | modus/mean | | SD | modus/mean | | SD | modus/mean | | SD |  |
| posture | 1.00 |  | 1.00 | |  | 3.00 | |  | 1.00 | |  | 1.00 | |  |  |
| muscular tonus | 0.00 |  | 0.00 | |  | 0.00 | |  | **-1.00^#^** | |  | 0.00 | |  |  |
| rearing | 10.8 | 3.4 | **4.6*** | | **3.6** | 7.4 | | 4.7 | **2.8*** | | **2.1** | **5.6*** | | **4.1** |  |
| hyperkinesis | 0.00 |  | **1.00*** | |  | 0.00 | |  | 0.00 | |  | 0.00 | |  |  |
| tremors | 0.00 |  | **1.00*** | |  | **1.00*** | |  | **0.00^#^** | |  | **0.00^#^** | |  |  |
| clonic movements | 0.00 |  | 2.00 | |  | 0.00 | |  | 0.00 | |  | 0.00 | |  |  |
| tonic movements | 0.00 |  | 0.00 | |  | 0.00 | |  | 0.00 | |  | 0.00 | |  |  |
| gait | 0.00 |  | **1.00*** | |  | **1.00*** | |  | **1.00*** | |  | **1.00*** | |  |  |
| ataxia | 0.00 |  | **1.00*** | |  | **1.00*** | |  | **1.00*** | |  | **1.00*** | |  |  |
| total disability score | 1.00 |  | 1.00 | |  | **2.00*^#^** | |  | 1.00 | |  | 1.00 | |  |  |
| mobility score | 1.00 |  | 1.00 | |  | 1.00 | |  | 1.00 | |  | 1.00 | |  |  |
| activity | 4.00 |  | 4.00 | |  | 4.00 | |  | 4.00 | |  | 4.00 | |  |  |
| air-righting reflex from the back position | 1.00 |  | 1.00 | |  | 1.00 | |  | 1.00 | |  | 1.00 | |  |  |
| air-righting reflex from the vertical position | 1.00 |  | **2.00*** | |  | **2.00*** | |  | **2.00*** | |  | **2.00*** | |  |  |
| landing foot splay (cm) | 12.8 | 1.9 | **9.8*** | | **2.7** | 11.3 | | 1.2 | 10.9 | | 2.6 | **12.1^#^** | | **1.4** |  |
| hindlimb grip strength (kg) | 3.82 | 0.71 | 3.90 | | 0.60 | **2.7*^#^** | | **0.33** | **2.49*^#^** | | **0.63** | 6.06 | | 8.47 |  |
| forelimb grip strength (kg) | 11.6 | 2.4 | **15.8*** | | **3.4** | 13.1 | | 2.2 | 13.2 | | 2.6 | 13.2 | | 3.3 |  |
| grip strength of all limbs (kg) | 16.3 | 6.4 | 22.3 | | 5.6 | 19.5 | | 3.9 | **15.3^#^** | | **2.7** | 20.1 | | 6.5 |  |
|  | **24 hours** | | | | | | | | | | | | | | |
| posture | 3.00 |  | **1.00*** | |  | **3.00^#^** | |  | **3.00^#^** | |  | **3.00^#^** | |  |  |
| muscular tonus | 0.00 |  | 0.00 | |  | 0.00 | |  | 0.00 | |  | 0.00 | |  |  |
| rearing | 2.7 | 2.3 | **15.6*** | | **9.0** | **7.6*^#^** | | **4.2** | **8.9*** | | **6.2** | **4.6^#^** | | **3.5** |  |
| hyperkinesis | 0.00 |  | **2.00*** | |  | **0.00^#^** | |  | **0.00^#^** | |  | **2.00*** | |  |  |
| tremors | 0.00 |  | 0.00 | |  | 0.00 | |  | 0.00 | |  | 0.00 | |  |  |
| clonic movements | 0.00 |  | **2.00*** | |  | 0.00 | |  | 0.00 | |  | **1.00*** | |  |  |
| tonic movements | 0.00 |  | 0.00 | |  | 0.00 | |  | 0.00 | |  | 0.00 | |  |  |
| gait | 0.00 |  | 0.00 | |  | 0.00 | |  | 0.00 | |  | 0.00 | |  |  |
| ataxia | 0.00 |  | 0.00 | |  | 0.00 | |  | 0.00 | |  | 0.00 | |  |  |
| total disability score | 1.00 |  | **2.00*** | |  | **1.00^#^** | |  | **1.00^#^** | |  | **2.00*** | |  |  |
| mobility score | 1.00 |  | 1.00 | |  | 1.00 | |  | 1.00 | |  | 1.00 | |  |  |
| activity | 3.00 |  | 1.00 | |  | **4.00^#^** | |  | **3.00^#^** | |  | **4.00^#^** | |  |  |
| air-righting reflex from the back position | 1.00 |  | 1.00 | |  | 1.00 | |  | 1.00 | |  | 1.00 | |  |  |
| air-righting reflex from the vertical position | 1.00 |  | 1.00 | |  | 1.00 | |  | 1.00 | |  | 1.00 | |  |  |
| landing foot splay (cm) | 12.0 | 1.0 | **9.8*** | | **1.9** | **8.2*** | | **1.5** | **8.5*** | | **1.7** | **10.5*** | | **1.4** |  |
| hindlimb grip strength (kg) | 4.37 | 1.47 | 4.82 | | 0.42 | **3.61^#^** | | **0.85** | **3.30^#^** | | **0.35** | 4.84 | | 1.54 |  |
| forelimb grip strength (kg) | 11.4 | 2.9 | 11.7 | | 2.9 | 12.2 | | 3.7 | 12.8 | | 2.3 | 11.4 | | 4.8 |  |
| grip strength of all limbs (kg) | 19.4 | 6.3 | 19.8 | | 3.3 | 22.2 | | 3.8 | 18.6 | | 2.9 | 19.1 | | 1.8 |  |

* Significantly different from the control group (saline-saline): p ≤ 0.05.

^#^ Significantly different from untreated A-234-intoxicated group (A-234-saline): p ≤ 0.05.
