# Supplementary material for: A-series agent A-234: initial in vitro and in vivo characterization
Source: Arch Toxicol. 2024 Mar 6;98(4):1135–49. doi: 10.1007/s00204-024-03689-3 (PMC10944400; doi:10.1007/s00204-024-03689-3)
Supplement: Supplementary file 17 — Supplementary file17 (DOCX 28 KB) [file 204_2024_3689_MOESM17_ESM.docx]

**Table S3.** Overview of sensory, motor, and excitability parameters assessed 2 and 24 h after the A-234 challenge (90% of LD_50_).

|  | **2 hours** | | | | | | | | | |
| --- | --- | --- | --- | --- | --- | --- | --- | --- | --- | --- |
|  | saline-saline | | A234-saline | | A234-atropine | | A234-atropine-methoxime | | A234- atropine-HI-6 | |
|  | modus/mean | SD | modus/mean | SD | modus/mean | SD | modus/mean | SD | modus/mean | SD |
| catch difficulty | 2.00 |  | **5.00*** |  | **2.00^#^** |  | **5.00*** |  | **5.00*** |  |
| ease of handling | 2.00 |  | 2.00 |  | 2.00 |  | **3.00*** |  | **3.00*** |  |
| tension | 0.00 |  | 0.00 |  | 0.00 |  | 0.00 |  | 0.00 |  |
| vocalisation | 0.00 |  | 0.00 |  | 0.00 |  | 0.00 |  | 0.00 |  |
| stereotypy | 0.00 |  | 0.00 |  | 0.00 |  | 0.00 |  | 0.00 |  |
| bizzare behavior | 0.00 |  | 0.00 |  | 0.00 |  | 0.00 |  | 0.00 |  |
| approach response | 1.00 |  | 1.00 |  | **2.00*^#^** |  | 1.00 |  | **2.00^#^** |  |
| touch response | 1.00 |  | **3.00*** |  | **2.00*^#^** |  | **3.00*** |  | **3.00*** |  |
| click response | 2.00 |  | 2.00 |  | 2.00 |  | **3.00*^#^** |  | 2.00 |  |
| tail-pinch response | 1.00 |  | 1.00 |  | **2.00*^#^** |  | **2.00*** |  | 1.00 |  |
|  | **24 hours** | | | | | | | | | |
| catch difficulty | 2.00 |  | 2.00 |  | 2.00 |  | 5.00 |  | 2.00 |  |
| ease of handling | 2.00 |  | 1.00 |  | 3.00 |  | 2.00 |  | 2.00 |  |
| tension | 0.00 |  | 0.00 |  | 0.00 |  | 0.00 |  | 0.00 |  |
| vocalisation | 0.00 |  | 0.00 |  | 0.00 |  | 0.00 |  | 0.00 |  |
| stereotypy | 0.00 |  | 0.00 |  | 0.00 |  | 0.00 |  | 0.00 |  |
| bizzare behavior | 0.00 |  | 0.00 |  | 0.00 |  | 0.00 |  | 0.00 |  |
| approach response | 1.00 |  | 1.00 |  | 1.00 |  | 1.00 |  | 1.00 |  |
| touch response | 1.00 |  | **3.00*** |  | **3.00*** |  | **3.00*** |  | **1.00^#^** |  |
| click response | 2.00 |  | **1.00*** |  | **3.00*** |  | 2.00 |  | 2.00 |  |
| tail-pinch response | 1.00 |  | 1.00 |  | 1.00 |  | **2.00*^#^** |  | 1.00 |  |

* Significantly different from the control group (saline-saline): p ≤ 0.05.

^#^ Significantly different from untreated A-234-intoxicated group (A-234-saline): p ≤ 0.05.
