# Supplementary material for: A-series agent A-234: initial in vitro and in vivo characterization
Source: Arch Toxicol. 2024 Mar 6;98(4):1135–49. doi: 10.1007/s00204-024-03689-3 (PMC10944400; doi:10.1007/s00204-024-03689-3)
Supplement: Supplementary file 18 — Supplementary file18 (DOCX 30 KB) [file 204_2024_3689_MOESM18_ESM.docx]

**Table S4.** Overview of vegetative parameters assessed 2 and 24 h after the A-234 challenge (90% of LD_50_).

|  | **2 hours** | | | | | | | | | |
| --- | --- | --- | --- | --- | --- | --- | --- | --- | --- | --- |
|  | saline-saline | | A234-saline | | A234-atropine | | A234-atropine-methoxime | | A234- atropine-HI-6 | |
|  | modus/mean | SD | modus/mean | SD | modus/mean | SD | modus/mean | SD | modus/mean | SD |
| lacrimation | 0.00 |  | 0.00 |  | 0.00 |  | 0.00 |  | 0.00 |  |
| lids position | 1.00 |  | 1.00 |  | 1.00 |  | 1.00 |  | 1.00 |  |
| endo/exophtalmus | 0.00 |  | 0.00 |  | 0.00 |  | 0.00 |  | 0.00 |  |
| fur abnormalities | 0.00 |  | **2.00*** |  | **2.00*** |  | **2.00*** |  | **2.00*** |  |
| skin abnormalities | 0.00 |  | 0.00 |  | 0.00 |  | 0.00 |  | 0.00 |  |
| salivation | 0.00 |  | 0.00 |  | 0.00 |  | 0.00 |  | 0.00 |  |
| nose secretion | 0.00 |  | 1.00 |  | **0.00^#^** |  | **0.00^#^** |  | **0.00^#^** |  |
| urination | 1.67 | 4.08 | 0.57 | 1.51 | **4.50^#^** | 4.84 | 1.25 | 3.54 | 0.00 | 0.00 |
| defecation | 0.00 | 0.00 | 0.00 | 0.00 | 0.50 | 0.93 | 0.50 | 1.07 | 0.00 | 0.00 |
| pupil size | 0.00 |  | **-2.00*** |  | **2.00*^#^** |  | **2.00*^#^** |  | **2.00*^#^** |  |
| pupil response | 1.00 |  | **0.00*** |  | **0.00*** |  | **0.00*** |  | **0.00*** |  |
| body weight (g) | 416.5 | 44.8 | 440.9 | 38.6 | 427.6 | 24.3 | 423.8 | 40.1 | 440.8 | 40.3 |
| respiration | 0.00 |  | **-1.00*** |  | **-1.00*** |  | **-1.00*** |  | **-1.00*** |  |
|  | **24 hours** | | | | | | | | | |
| lacrimation | 0.00 |  | 0.00 |  | 0.00 |  | 0.00 |  | 0.00 |  |
| lids position | 1.00 |  | 1.00 |  | 1.00 |  | 1.00 |  | 1.00 |  |
| endo/exophtalmus | 0.00 |  | 0.00 |  | 0.00 |  | 0.00 |  | 0.00 |  |
| fur abnormalities | 0.00 |  | 0.00 |  | 0.00 |  | **2.00*^#^** |  | **2.00*^#^** |  |
| skin abnormalities | 0.00 |  | 0.00 |  | 0.00 |  | 0.00 |  | 0.00 |  |
| salivation | 0.00 |  | 0.00 |  | 0.00 |  | 0.00 |  | 0.00 |  |
| nose secretion | 0.00 |  | 0.00 |  | 0.00 |  | 0.00 |  | 0.00 |  |
| urination | 0.00 | 0.00 | 0.20 | 0.45 | 0.00 | 0.00 | 0.00 | 0.00 | 1.25 | 3.54 |
| defecation | 0.50 | 1.22 | 0.80 | 1.30 | 0.13 | 0.35 | 0.38 | 0.52 | 1.00 | 1.60 |
| pupil size | 0.00 |  | 0.00 |  | 0.00 |  | 0.00 |  | 0.00 |  |
| pupil response | 1.00 |  | 1.00 |  | 1.00 |  | 1.00 |  | 1.00 |  |
| body weight (g) | 415.0 | 40.9 | 443.4 | 23.5 | 419.6 | 24.0 | 415.5 | 37.6 | 431.6 | 20.1 |
| respiration | 0.00 |  | 0.00 |  | 0.00 |  | 0.00 |  | 0.00 |  |

* Significantly different from the control group (saline-saline): p ≤ 0.05.

^#^ Significantly different from untreated A-234-intoxicated group (A-234-saline): p ≤ 0.05.
