# Supplementary material for: A-series agent A-234: initial in vitro and in vivo characterization
Source: Arch Toxicol. 2024 Mar 6;98(4):1135–49. doi: 10.1007/s00204-024-03689-3 (PMC10944400; doi:10.1007/s00204-024-03689-3)
Supplement: Supplementary file 19 — Supplementary file19 (DOCX 1316 KB) [file 204_2024_3689_MOESM19_ESM.docx]

**Supplementary Information**

**A-series agent A-234: Initial *in vitro* and *in vivo* characterization**

Martina Hrabinova^a‡^, Jaroslav Pejchal^a‡^, Vendula Hepnarova^a,*^, Lubica Muckova^a,b^, Lucie Junova^a,b^, Jakub Opravil^a^, Jana Zdarova Karasova^a,b^, Tomas Rozsypal^c^, Alzbeta Dlabkova^a^, Helena Rehulkova^a^, Tomas Kucera^d^, Zbyněk Vecera^a^, Filip Caisberger^e^, Monika Schmidt^b,f^, Ondrej Soukup*^b^*, Daniel Jun^a^*

*^a^ University of Defence, Faculty of Military Health Sciences, Department of Toxicology and Military Pharmacy, Trebesska 1575, 500 01 Hradec Kralove, Czech Republic*

*^b^ University Hospital Hradec Kralove, Biomedical Research Centre, Sokolska 581, 500 05 Hradec Kralove, Czech Republic*

*^c^ Nuclear, Biological and Chemical Defence Institute, University of Defence, Vita Nejedleho 1, 68203* *Vyskov, Czech Republic*

*^d^ University of Defence, Faculty of Military Health Sciences, Department of Military Medical Service Organization and Management, Trebesska 1575, 500 01 Hradec Kralove, Czech Republic*

*^e^ University Hospital Hradec Kralove, Department of Neurology, Sokolska 581, 500 05 Hradec Kralove, Czech Republic*

*^f^ University Hradec Kralove, Faculty of Science, Department of Chemistry, Rokitanskeho 62, 50003 Hradec Kralove, Czech Republic*

**^‡^** These authors contributed equally

* Corresponding author:

Vendula Hepnarova (vendula.hepnarova@unob.cz), University of Defence, Faculty of Military Health Sciences, Department of Toxicology and Military Pharmacy, Trebesska 1575, 500 01 Hradec Kralove, Czech Republic; Tel.: + 420 973255172; ORCID: [0000-0002-6445-2669](https://orcid.org/0000-0002-6445-2669)

Daniel Jun (daniel.jun@unob.cz), University of Defence, Faculty of Military Health Sciences, Department of Toxicology and Military Pharmacy, Trebesska 1575, 500 01 Hradec Kralove, Czech Republic; Tel.: + 420 973 255 150; ORCID: [0000-0002-0882-6304](https://orcid.org/0000-0002-0882-6304)

**Table of contents**

[1. Stability of A-234 in 2-propanol 2](#_Toc153365628)

[2. Half-life measurement 3](#_Toc153365629)

[3. Reactivation kinetics measurement 5](#_Toc153365630)

[4. Functional observatory battery (FOB) 6](#_Toc153365631)

[5. Molecular dynamics 14](#_Toc153365632)

# Stability of A-234 in 2-propanol

A 234 (Military Research Institute, s. e., Brno, Czech Republic) from a freshly opened vial (batch purity 99.0% ) was dissolved in an anhydrous 2-propanol (99.5%, Merck, Darmstadt, Germany) to a concentration of 50 µg/mL and left at a laboratory temperature. The quantitative analysis was performed by gas chromatography-tandem mass spectrometry (GC-MS) using GC-MS system Agilent 7000D GC/QQQ (Agilent Technologies, Santa Clara, CA, USA) each 23.5 min at a time range from 0 to 1175 min. An HP-5ms Ultra Inert column (30 m, 0.25 mm, 0.25 μm; Agilent Technologies) was used as the stationary phase and helium as a carrier gas. The injector was operated in split mode at 250 °C. The injected volume was 1 µL. The oven temperature was maintained at 60 °C for 2 minutes, then increased to 280 °C (20 °C/min) and was held for 6 minutes. The mass spectrometer was operated with electron ionization in full scan mode. The temperature of the ion source was 230 °C.

The retention time of A234 was 9.7 min. The ions m/z 224, 195, 167, 152, 124, 68, and 42 were dominant in the A234 spectrum. The values in the graph indicate % quantity when the initial time point (0 min) concentration was considered 100%. The graph was done using GraphPad Prism version 9.5.1 for Windows.


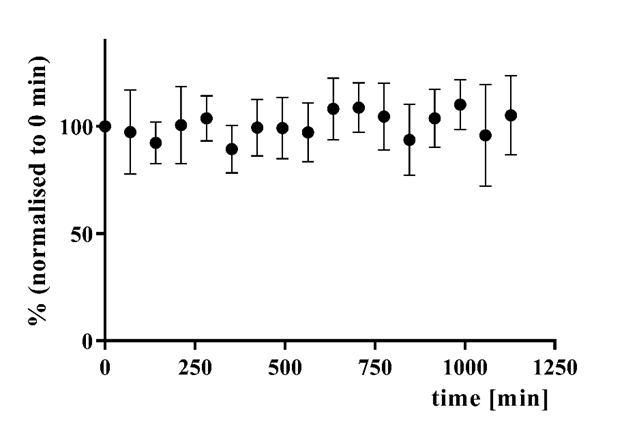


**Fig. S1.** Stability of A234 in propan-2-ol: graph summarizing averaged quantified results normalized to 0 min ± standard deviation, n = 5.

# Half-life measurement

Modified Ellman’s protocol was used to investigate the inhibition half-life, i.e., the time when the enzyme activity is reduced by given concentration by 50%, of nerve agents towards *Hss*AChE and *Hss*BChE (Ellman et al. 1961). Polystyrene Nunc 96-well microplates with a flat bottom shape (Thermo Fisher Scientific) were utilized. The assay medium consisted of 40 µL of 0.1 M phosphate buffer (pH 7.4), 20 µL of 0.01 M DTNB, 10 µL of the enzyme, 10 µL of OP solution, and 20 µL of 0.01 M (ATChI/BTChI). and 20 µL of 0.01 M substrate (acetylthiocholine [ATCh]/butyrylthiocholine [BTCh]; both from Merck). Organophosphate solutions with A-234, sarin, or VX (batches purity 99.0 %, 99.0 %, and 93.5 %, respectively, all from Military Research Institute) were prepared in propan-2-ol.

First, the IC_50_ value (in 5 min) was assessed. This concentration was then used for the time-dependent measurement. The enzyme activity was determined by measuring the change in absorbance at 412 nm at 37 °C at 5 min intervals in 1 hour. The calculations (one phase decay function) and graphs for half-life were done using GraphPad Prism version 9.5.1 for Windows. Further, IC_50_ value (in 5× half-life) was measured and calculated for each enzyme. According to the timeline of an exponential decay process, five inhibition half-lives will cause approx. 97% inhibition of the enzyme, which is optimal for follow-up inhibition/reactivation assay (Hacker et al. 2009). A representative figure of half-life measurement for sarin is depicted in Fig. S1.


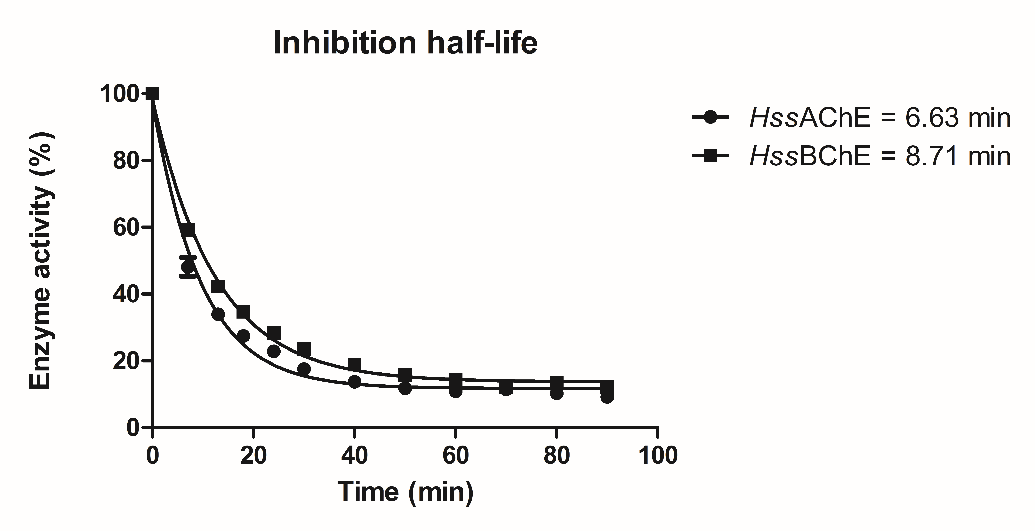


**Fig. S2.** The time course of enzyme activity inhibited by sarin at concentrations 10^-4.75^ and
10^-4.5^ M for *Hss*AChE and *Hss*BChE, respectively.

# Reactivation kinetics measurement


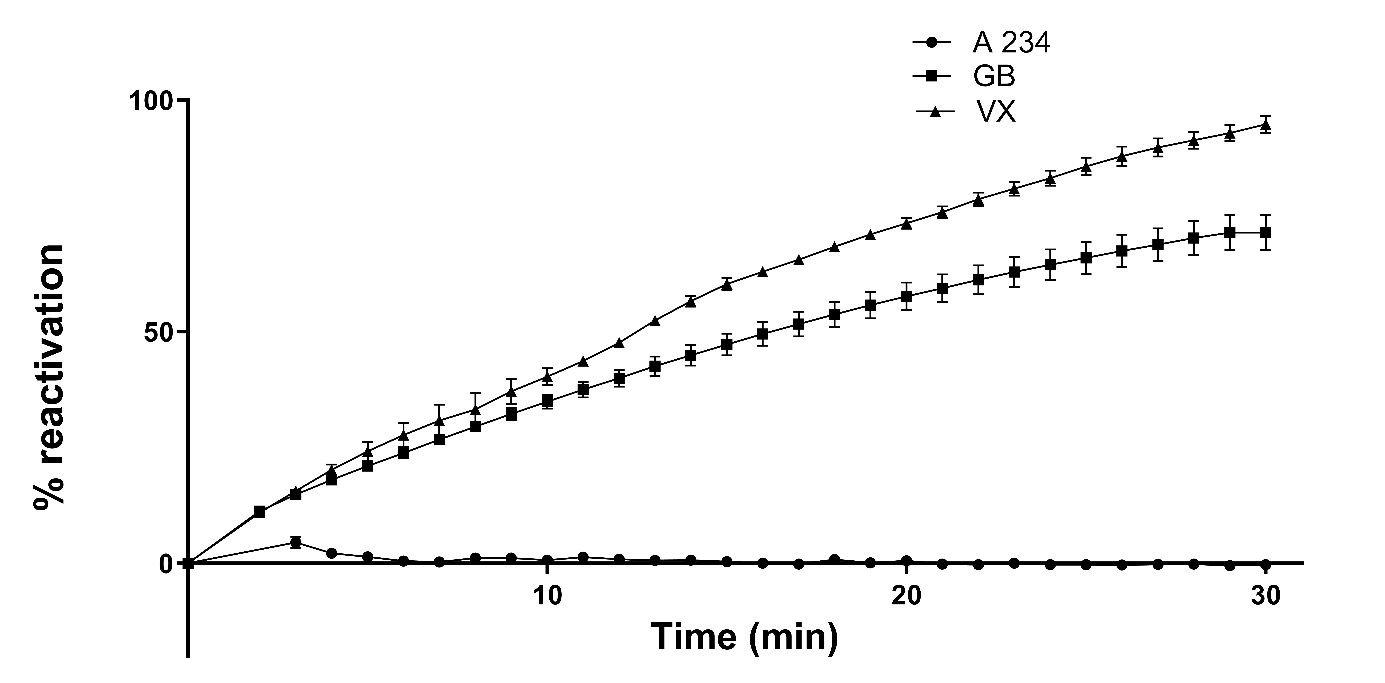


**Fig. S3**. The time course of reactivation of *Hss*AChE inhibited by A-234, GB, and VX by 0.025 mM HI-6.


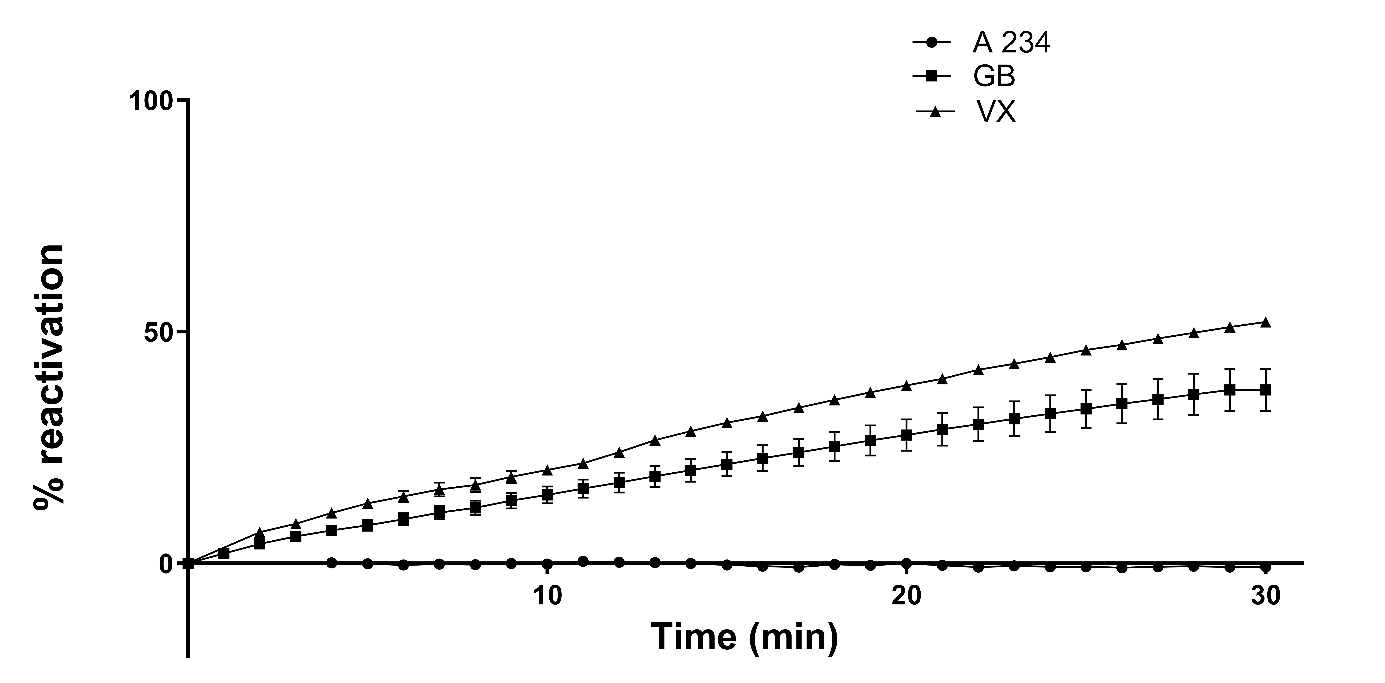


**Fig. S4.**  The time course of reactivation of *Hss*AChE inhibited by A-234, GB, and VX by 0.01 mM HI-6.

# Functional observatory battery (FOB)

**Table S1**. Signs and symptoms assessed in the functional observatory battery and their semi-quantitative scales.

| **Marker** | **Scored values** | | | | | | | | | |
| --- | --- | --- | --- | --- | --- | --- | --- | --- | --- | --- |
|  | **-2** | **-1** | **0** | **1** | **2** | **3** | **4** | **5** | **6** | **7** |
| **posture** |  |  |  | ***sitting or standing*** | ***rearing*** | ***asleep*** | flattened | lying on side | crouched over | head bobbing |
| **muscular tonus** | atonic | hypotonic | ***normal*** | hypertonic | rigidity | fasciculation |  |  |  |  |
| **hyperkinesis** |  |  | ***normal***  ***kinesis*** | repetitive movements of mouth and jaws | non-rhythmic quivers | mild tremors | severe tremors | myoclonic jerks | clonic convulsion |  |
| **tremors** |  |  | ***none*** | after stimulation | mild local | mild overall | medium–influencing movement | strong–impeding movement |  |  |
| **clonic movements** |  |  | ***none*** | twitches | non-rhythmic movement |  |  |  |  |  |
| **tonic movements** |  |  | ***normal*** | contraction of extensors | opistotonus | emprostotonus | explosive jumps | tonic convulsions |  |  |
| **gait** |  |  | ***normal*** | ataxia | overcompensation of hind limb movements | feet point outwards from the body | forelimbs are extended | walks on tiptoes | hunched body | the body is flattened against the surface |
| **ataxia** |  |  | ***none*** | mild | severe |  |  |  |  |  |
| **total disability score** |  |  |  | ***normal*** | slightly impaired | somewhat impaired | totally impaired |  |  |  |
| **mobility score** |  |  |  | ***normal*** | slightly impaired | somewhat impaired | totally impaired |  |  |  |
| **activity** |  |  |  | very low | sporadic | reduced | ***normal*** | enhanced | permanent |  |
| **righting reflex (from vertical or back position)** |  |  |  | ***normal*** | slightly uncoordinated | lands on side | lands on back |  |  |  |
| **catch difficulty** |  |  |  | passive | ***normal*** | defense | flight | escape | aggression |  |
| **ease of handling** |  |  |  | very easy | ***easy*** | moderately difficult | difficult |  |  |  |
| **tension** |  |  | ***none*** | partial (ears) | stupor |  |  |  |  |  |
| **vocalization** |  |  | ***none*** | provoked | spontaneous | excessive |  |  |  |  |
| **stereotypy** |  |  | ***none*** | head weaving | body weaving | grooming | circling | others |  |  |
| **bizarre behavior** |  |  | ***none*** | head | body | self-mutilation | abnormal movements | others |  |  |
| **approach response** |  |  |  | no reaction | ***normal*** | slow reaction | energetic reaction | exaggerated reaction |  |  |
| **touch response** |  |  |  | no reaction | ***normal*** | slow reaction | energetic reaction | exaggerated reaction |  |  |
| **click response** |  |  |  | no reaction | ***normal*** | slow reaction | energetic reaction | exaggerated reaction |  |  |
| **tail-pinch response** |  |  |  | no reaction | ***normal*** | slow reaction | energetic reaction | exaggerated reaction |  |  |
| **lacrimation** |  |  | ***none*** | slight | severe | crusts | colored crusts |  |  |  |
| **lids position** |  |  |  | ***open*** | slightly dropping | half-way dropping | completely shut | ptosis |  |  |
| **endo/exophthalmos** |  | enophthalmos | ***normal*** | exophthalmos |  |  |  |  |  |  |
| **fur abnormalities** |  |  | ***normal*** | colored | disheveled | colored and disheveled | baldness | injury | other changes | piloerection |
| **skin abnormalities** |  |  | ***normal*** | pale | erythema | cyanosis | pigmentation | cold | injury |  |
| **salivation** |  |  | ***none*** | slight | severe |  |  |  |  |  |
| **nose secretion** |  |  | ***none*** | slight | severe | colored |  |  |  |  |
| **pupil size** |  | miosis | ***normal*** | mydriasis |  |  |  |  |  |  |
| **pupil response** |  |  | no reaction | ***normal reaction*** |  |  |  |  |  |  |
| **respiration** | apnea | bradypnea | ***normal*** | tachypnea | dyspnea |  |  |  |  |  |

Signs and symptoms described in Bold italics represent the physiological range.

Symptoms, such as rearing (per min), urination (area of urine drops on absorbent paper), and defecation (number of poops), were directly quantified, the quantification period lasted for 3 min.

Landing foot splay (cm) and body weight (g) were measured by ruler and weight (A&D company, Tokyo, Japan), respectively.

Hindlimb, forelimb grip strength, and strength of all limbs were measured using a strength meter (Electronika, Praha, ČR).

**Table S2.** Overview of activity and neuro-muscular parameters assessed 2 and 24 h after the A-234 challenge (90% of LD_50_).

|  | 2 hours | | | | | | | | | | | | | | |
| --- | --- | --- | --- | --- | --- | --- | --- | --- | --- | --- | --- | --- | --- | --- | --- |
|  | saline-saline | | | A234-saline | | | A234-atropine | | | A234-atropine-methoxime | | | A234- atropine-HI-6 | | |
|  | modus/mean | SD | modus/mean | | SD | modus/mean | | SD | modus/mean | | SD | modus/mean | | SD |  |
| posture | 1.00 |  | 1.00 | |  | 3.00 | |  | 1.00 | |  | 1.00 | |  |  |
| muscular tonus | 0.00 |  | 0.00 | |  | 0.00 | |  | **-1.00^#^** | |  | 0.00 | |  |  |
| rearing | 10.8 | 3.4 | **4.6*** | | **3.6** | 7.4 | | 4.7 | **2.8*** | | **2.1** | **5.6*** | | **4.1** |  |
| hyperkinesis | 0.00 |  | **1.00*** | |  | 0.00 | |  | 0.00 | |  | 0.00 | |  |  |
| tremors | 0.00 |  | **1.00*** | |  | **1.00*** | |  | **0.00^#^** | |  | **0.00^#^** | |  |  |
| clonic movements | 0.00 |  | 2.00 | |  | 0.00 | |  | 0.00 | |  | 0.00 | |  |  |
| tonic movements | 0.00 |  | 0.00 | |  | 0.00 | |  | 0.00 | |  | 0.00 | |  |  |
| gait | 0.00 |  | **1.00*** | |  | **1.00*** | |  | **1.00*** | |  | **1.00*** | |  |  |
| ataxia | 0.00 |  | **1.00*** | |  | **1.00*** | |  | **1.00*** | |  | **1.00*** | |  |  |
| total disability score | 1.00 |  | 1.00 | |  | **2.00*^#^** | |  | 1.00 | |  | 1.00 | |  |  |
| mobility score | 1.00 |  | 1.00 | |  | 1.00 | |  | 1.00 | |  | 1.00 | |  |  |
| activity | 4.00 |  | 4.00 | |  | 4.00 | |  | 4.00 | |  | 4.00 | |  |  |
| air-righting reflex from the back position | 1.00 |  | 1.00 | |  | 1.00 | |  | 1.00 | |  | 1.00 | |  |  |
| air-righting reflex from the vertical position | 1.00 |  | **2.00*** | |  | **2.00*** | |  | **2.00*** | |  | **2.00*** | |  |  |
| landing foot splay (cm) | 12.8 | 1.9 | **9.8*** | | **2.7** | 11.3 | | 1.2 | 10.9 | | 2.6 | **12.1^#^** | | **1.4** |  |
| hindlimb grip strength (kg) | 3.82 | 0.71 | 3.90 | | 0.60 | **2.7*^#^** | | **0.33** | **2.49*^#^** | | **0.63** | 6.06 | | 8.47 |  |
| forelimb grip strength (kg) | 11.6 | 2.4 | **15.8*** | | **3.4** | 13.1 | | 2.2 | 13.2 | | 2.6 | 13.2 | | 3.3 |  |
| grip strength of all limbs (kg) | 16.3 | 6.4 | 22.3 | | 5.6 | 19.5 | | 3.9 | **15.3^#^** | | **2.7** | 20.1 | | 6.5 |  |
|  | **24 hours** | | | | | | | | | | | | | | |
| posture | 3.00 |  | **1.00*** | |  | **3.00^#^** | |  | **3.00^#^** | |  | **3.00^#^** | |  |  |
| muscular tonus | 0.00 |  | 0.00 | |  | 0.00 | |  | 0.00 | |  | 0.00 | |  |  |
| rearing | 2.7 | 2.3 | **15.6*** | | **9.0** | **7.6*^#^** | | **4.2** | **8.9*** | | **6.2** | **4.6^#^** | | **3.5** |  |
| hyperkinesis | 0.00 |  | **2.00*** | |  | **0.00^#^** | |  | **0.00^#^** | |  | **2.00*** | |  |  |
| tremors | 0.00 |  | 0.00 | |  | 0.00 | |  | 0.00 | |  | 0.00 | |  |  |
| clonic movements | 0.00 |  | **2.00*** | |  | 0.00 | |  | 0.00 | |  | **1.00*** | |  |  |
| tonic movements | 0.00 |  | 0.00 | |  | 0.00 | |  | 0.00 | |  | 0.00 | |  |  |
| gait | 0.00 |  | 0.00 | |  | 0.00 | |  | 0.00 | |  | 0.00 | |  |  |
| ataxia | 0.00 |  | 0.00 | |  | 0.00 | |  | 0.00 | |  | 0.00 | |  |  |
| total disability score | 1.00 |  | **2.00*** | |  | **1.00^#^** | |  | **1.00^#^** | |  | **2.00*** | |  |  |
| mobility score | 1.00 |  | 1.00 | |  | 1.00 | |  | 1.00 | |  | 1.00 | |  |  |
| activity | 3.00 |  | 1.00 | |  | **4.00^#^** | |  | **3.00^#^** | |  | **4.00^#^** | |  |  |
| air-righting reflex from the back position | 1.00 |  | 1.00 | |  | 1.00 | |  | 1.00 | |  | 1.00 | |  |  |
| air-righting reflex from the vertical position | 1.00 |  | 1.00 | |  | 1.00 | |  | 1.00 | |  | 1.00 | |  |  |
| landing foot splay (cm) | 12.0 | 1.0 | **9.8*** | | **1.9** | **8.2*** | | **1.5** | **8.5*** | | **1.7** | **10.5*** | | **1.4** |  |
| hindlimb grip strength (kg) | 4.37 | 1.47 | 4.82 | | 0.42 | **3.61^#^** | | **0.85** | **3.30^#^** | | **0.35** | 4.84 | | 1.54 |  |
| forelimb grip strength (kg) | 11.4 | 2.9 | 11.7 | | 2.9 | 12.2 | | 3.7 | 12.8 | | 2.3 | 11.4 | | 4.8 |  |
| grip strength of all limbs (kg) | 19.4 | 6.3 | 19.8 | | 3.3 | 22.2 | | 3.8 | 18.6 | | 2.9 | 19.1 | | 1.8 |  |

* Significantly different from the control group (saline-saline): p ≤ 0.05.

^#^ Significantly different from untreated A-234-intoxicated group (A-234-saline): p ≤ 0.05.

**Table S3.** Overview of sensory, motor, and excitability parameters assessed 2 and 24 h after the A-234 challenge (90% of LD_50_).

|  | **2 hours** | | | | | | | | | |
| --- | --- | --- | --- | --- | --- | --- | --- | --- | --- | --- |
|  | saline-saline | | A234-saline | | A234-atropine | | A234-atropine-methoxime | | A234- atropine-HI-6 | |
|  | modus/mean | SD | modus/mean | SD | modus/mean | SD | modus/mean | SD | modus/mean | SD |
| catch difficulty | 2.00 |  | **5.00*** |  | **2.00^#^** |  | **5.00*** |  | **5.00*** |  |
| ease of handling | 2.00 |  | 2.00 |  | 2.00 |  | **3.00*** |  | **3.00*** |  |
| tension | 0.00 |  | 0.00 |  | 0.00 |  | 0.00 |  | 0.00 |  |
| vocalisation | 0.00 |  | 0.00 |  | 0.00 |  | 0.00 |  | 0.00 |  |
| stereotypy | 0.00 |  | 0.00 |  | 0.00 |  | 0.00 |  | 0.00 |  |
| bizzare behavior | 0.00 |  | 0.00 |  | 0.00 |  | 0.00 |  | 0.00 |  |
| approach response | 1.00 |  | 1.00 |  | **2.00*^#^** |  | 1.00 |  | **2.00^#^** |  |
| touch response | 1.00 |  | **3.00*** |  | **2.00*^#^** |  | **3.00*** |  | **3.00*** |  |
| click response | 2.00 |  | 2.00 |  | 2.00 |  | **3.00*^#^** |  | 2.00 |  |
| tail-pinch response | 1.00 |  | 1.00 |  | **2.00*^#^** |  | **2.00*** |  | 1.00 |  |
|  | **24 hours** | | | | | | | | | |
| catch difficulty | 2.00 |  | 2.00 |  | 2.00 |  | 5.00 |  | 2.00 |  |
| ease of handling | 2.00 |  | 1.00 |  | 3.00 |  | 2.00 |  | 2.00 |  |
| tension | 0.00 |  | 0.00 |  | 0.00 |  | 0.00 |  | 0.00 |  |
| vocalisation | 0.00 |  | 0.00 |  | 0.00 |  | 0.00 |  | 0.00 |  |
| stereotypy | 0.00 |  | 0.00 |  | 0.00 |  | 0.00 |  | 0.00 |  |
| bizzare behavior | 0.00 |  | 0.00 |  | 0.00 |  | 0.00 |  | 0.00 |  |
| approach response | 1.00 |  | 1.00 |  | 1.00 |  | 1.00 |  | 1.00 |  |
| touch response | 1.00 |  | **3.00*** |  | **3.00*** |  | **3.00*** |  | **1.00^#^** |  |
| click response | 2.00 |  | **1.00*** |  | **3.00*** |  | 2.00 |  | 2.00 |  |
| tail-pinch response | 1.00 |  | 1.00 |  | 1.00 |  | **2.00*^#^** |  | 1.00 |  |

* Significantly different from the control group (saline-saline): p ≤ 0.05.

^#^ Significantly different from untreated A-234-intoxicated group (A-234-saline): p ≤ 0.05.

**Table S4.** Overview of vegetative parameters assessed 2 and 24 h after the A-234 challenge (90% of LD_50_).

|  | **2 hours** | | | | | | | | | |
| --- | --- | --- | --- | --- | --- | --- | --- | --- | --- | --- |
|  | saline-saline | | A234-saline | | A234-atropine | | A234-atropine-methoxime | | A234- atropine-HI-6 | |
|  | modus/mean | SD | modus/mean | SD | modus/mean | SD | modus/mean | SD | modus/mean | SD |
| lacrimation | 0.00 |  | 0.00 |  | 0.00 |  | 0.00 |  | 0.00 |  |
| lids position | 1.00 |  | 1.00 |  | 1.00 |  | 1.00 |  | 1.00 |  |
| endo/exophtalmus | 0.00 |  | 0.00 |  | 0.00 |  | 0.00 |  | 0.00 |  |
| fur abnormalities | 0.00 |  | **2.00*** |  | **2.00*** |  | **2.00*** |  | **2.00*** |  |
| skin abnormalities | 0.00 |  | 0.00 |  | 0.00 |  | 0.00 |  | 0.00 |  |
| salivation | 0.00 |  | 0.00 |  | 0.00 |  | 0.00 |  | 0.00 |  |
| nose secretion | 0.00 |  | 1.00 |  | **0.00^#^** |  | **0.00^#^** |  | **0.00^#^** |  |
| urination | 1.67 | 4.08 | 0.57 | 1.51 | **4.50^#^** | 4.84 | 1.25 | 3.54 | 0.00 | 0.00 |
| defecation | 0.00 | 0.00 | 0.00 | 0.00 | 0.50 | 0.93 | 0.50 | 1.07 | 0.00 | 0.00 |
| pupil size | 0.00 |  | **-2.00*** |  | **2.00*^#^** |  | **2.00*^#^** |  | **2.00*^#^** |  |
| pupil response | 1.00 |  | **0.00*** |  | **0.00*** |  | **0.00*** |  | **0.00*** |  |
| body weight (g) | 416.5 | 44.8 | 440.9 | 38.6 | 427.6 | 24.3 | 423.8 | 40.1 | 440.8 | 40.3 |
| respiration | 0.00 |  | **-1.00*** |  | **-1.00*** |  | **-1.00*** |  | **-1.00*** |  |
|  | **24 hours** | | | | | | | | | |
| lacrimation | 0.00 |  | 0.00 |  | 0.00 |  | 0.00 |  | 0.00 |  |
| lids position | 1.00 |  | 1.00 |  | 1.00 |  | 1.00 |  | 1.00 |  |
| endo/exophtalmus | 0.00 |  | 0.00 |  | 0.00 |  | 0.00 |  | 0.00 |  |
| fur abnormalities | 0.00 |  | 0.00 |  | 0.00 |  | **2.00*^#^** |  | **2.00*^#^** |  |
| skin abnormalities | 0.00 |  | 0.00 |  | 0.00 |  | 0.00 |  | 0.00 |  |
| salivation | 0.00 |  | 0.00 |  | 0.00 |  | 0.00 |  | 0.00 |  |
| nose secretion | 0.00 |  | 0.00 |  | 0.00 |  | 0.00 |  | 0.00 |  |
| urination | 0.00 | 0.00 | 0.20 | 0.45 | 0.00 | 0.00 | 0.00 | 0.00 | 1.25 | 3.54 |
| defecation | 0.50 | 1.22 | 0.80 | 1.30 | 0.13 | 0.35 | 0.38 | 0.52 | 1.00 | 1.60 |
| pupil size | 0.00 |  | 0.00 |  | 0.00 |  | 0.00 |  | 0.00 |  |
| pupil response | 1.00 |  | 1.00 |  | 1.00 |  | 1.00 |  | 1.00 |  |
| body weight (g) | 415.0 | 40.9 | 443.4 | 23.5 | 419.6 | 24.0 | 415.5 | 37.6 | 431.6 | 20.1 |
| respiration | 0.00 |  | 0.00 |  | 0.00 |  | 0.00 |  | 0.00 |  |

* Significantly different from the control group (saline-saline): p ≤ 0.05.

^#^ Significantly different from untreated A-234-intoxicated group (A-234-saline): p ≤ 0.05.

# Molecular dynamics

**
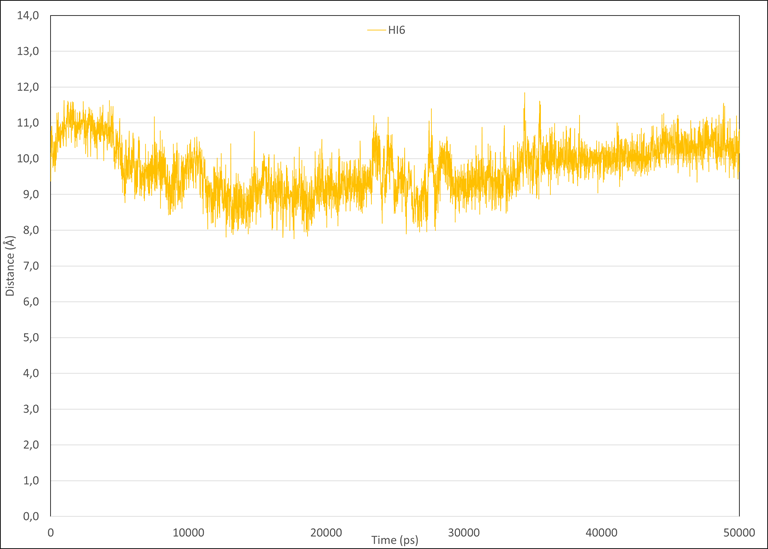
**

**Fig. S5.** Results of 50-ns molecular dynamics simulation showing the distance between HI-6 oxygen and A-234 phosphorus analyzed by the Visual Molecular Dynamics software version 1.9.3.

**
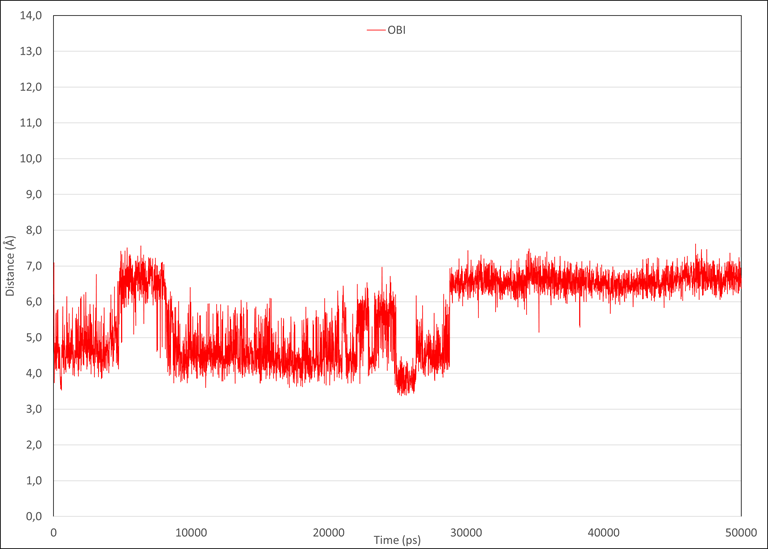
**

**Fig. S6.** Results of 50-ns molecular dynamics simulation showing the distance between obidoxime oxygen and A-234 phosphorus analyzed by the Visual Molecular Dynamics software version 1.9.3.

**
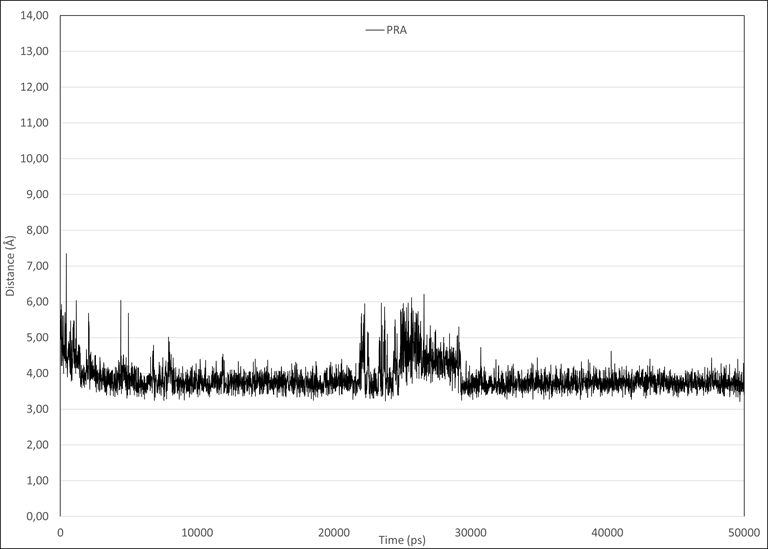
**

**Fig. S7.** Results of 50-ns molecular dynamics simulation showing the distance between pralidoxime oxygen and A-234 phosphorus analyzed by the Visual Molecular Dynamics software version 1.9.3.

**
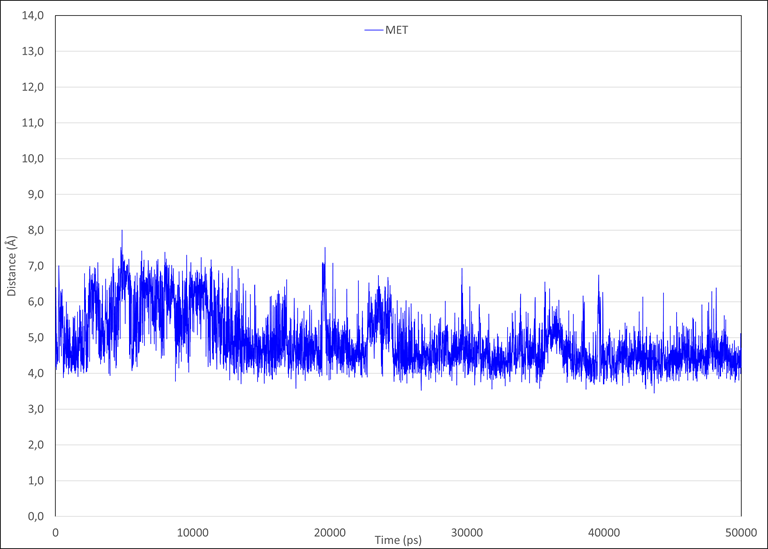
**

**Fig. S8.** Results of 50-ns molecular dynamics simulation showing the distance between methoxime oxygen and A-234 phosphorus analyzed by the Visual Molecular Dynamics software version 1.9.3.

**
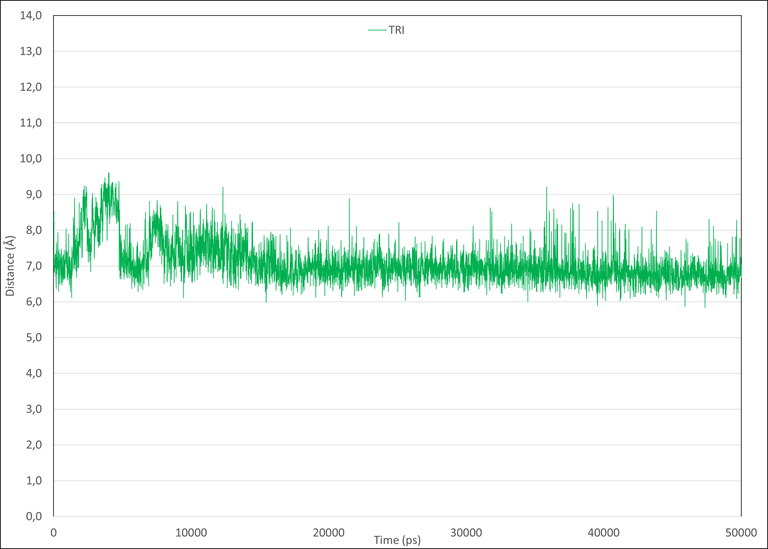
**

**Fig. S9.** Results of 50-ns molecular dynamics simulation showing the distance between trimedoxime oxygen and A-234 phosphorus analyzed by the Visual Molecular Dynamics software version 1.9.3.

**
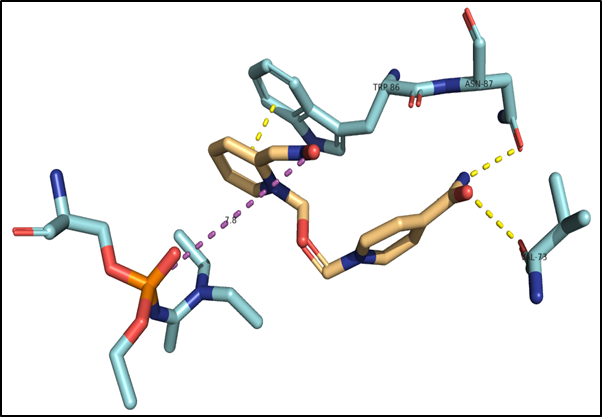
**

**Fig. S10.** 3D representation of HI-6- inhibited HssAChE interaction. The distance between the oxygen of the oxime group and A-234 phosphorus is 7.75 Å. For HI-6, we observed significant π-π stacking between its aromatic part and residue Trp86. HI-6 carbamoyl moiety also forms hydrogen bonds with residues VAL73 and ASN87.

**
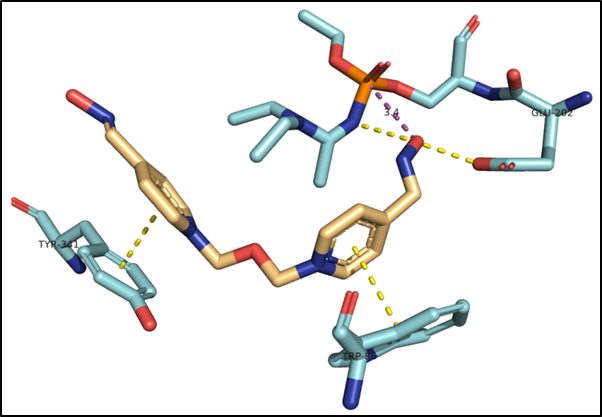
**

**Fig. S11.** 3D representation of obidoxime-inhibited *Hss*AChE interaction. The distance between the oxygen of the oxime group and A-234 phosphorus is 3.37 Å. For obidoxime, we observed significant π-π stacking between its aromatic part and residues Trp86 and Tyr341. Its oxime moiety also forms a hydrogen bond with residue Glu202.

**
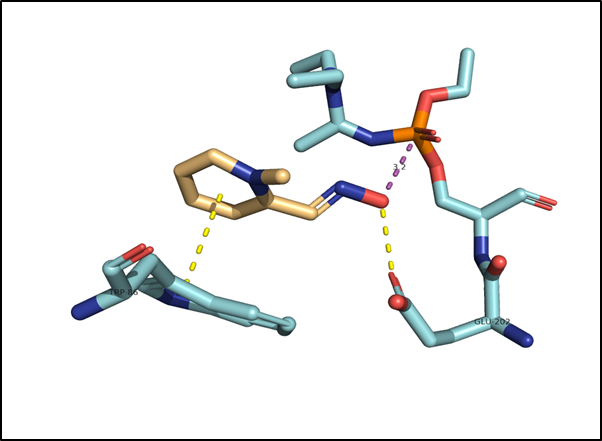
**

**Fig. S12.** 3D representation of pralidoxime-inhibited *Hss*AChE interaction. The distance between the oxygen of the oxime group and A-234 phosphorus is 3.21 Å. For pralidoxime, we observed significant π-π stacking between its aromatic part and residue Trp86. Its oxime moiety forms a hydrogen bond with residue Glu202.

**
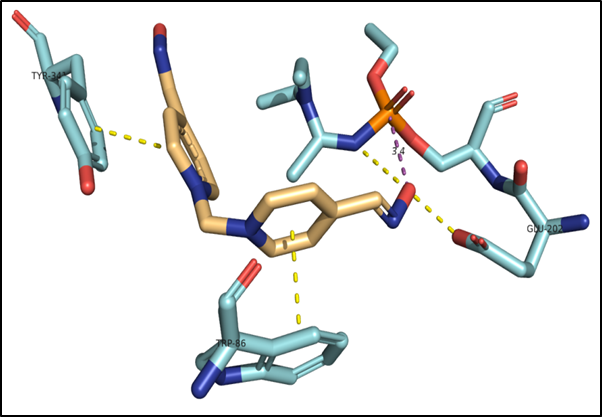
**

**Fig. S13.** 3D representation of methoxime-inhibited *Hss*AChE interaction. The distance between the oxygen of the oxime group and A-234 phosphorus is 3.44 Å. For methoxime, we observed significant π-π stacking between its aromatic part and residues Trp86 and Tyr341. Its oxime moiety forms a hydrogen bond with residue Glu202.

**
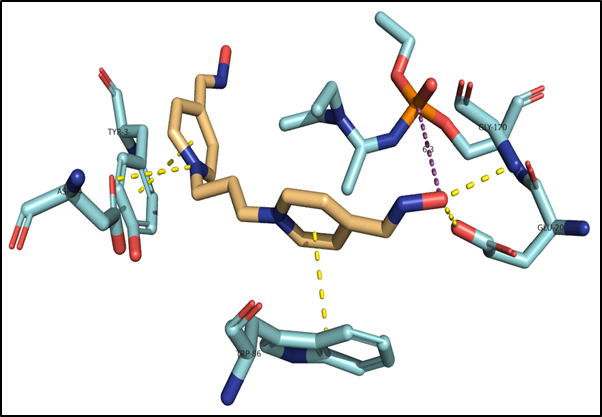
**

**Fig. S14.** 3D representation of trimedoxime-inhibited HssAChE interaction. The distance between the oxygen of the oxime group and A-234 phosphorus is 6.27Å. For trimedoxime, we observed significant π-π stacking between its aromatic part and residues Trp86 and Tyr341. Its oxime moiety forms hydrogen bonds with residues Gly120 and Glu202. Additionally, its pyridinium nitrogen forms a hydrogen bond with residue Asp74.
